# Mental health multimorbidity and physical conditions in children aged 6–17 years in the United States

**DOI:** 10.1002/gps3.70048

**Published:** 2026-08-17

**Authors:** Hua He, Feng Li, Guodong Ding, Fei Li

**Affiliations:** ^1^ Developmental and Behavioral Pediatric Department & Child Primary Care Department Xinhua Hospital Affiliated to Shanghai Jiao Tong University School of Medicine Shanghai China; ^2^ Ministry of Education‐Shanghai Key Laboratory of Children's Environmental Health Xinhua Hospital Affiliated to Shanghai Jiao Tong University School of Medicine Shanghai China; ^3^ Department of Pediatrics Xinhua Hospital Affiliated to Shanghai Jiao Tong University School of Medicine Shanghai China

**Keywords:** children and adolescents, dose‐response relationship, latent class analysis, mental health multimorbidity, physical condition

## Abstract

**Background:**

The rising prevalence and early onset of mental health problems among children and adolescents pose a critical public health challenge.

**Aims:**

This study aimed to identify distinct profiles of mental health multimorbidity, examine their associations with physical conditions and evaluate the dose–response relationships between the number of mental health multimorbidities and physical condition risk.

**Methods:**

Using cross‐sectional data from 30 556 children aged 6–17 years in the 2023 United States National Survey of Children's Health, we applied latent class analysis to derive multimorbidity profiles from nine mental health problems. Multivariable logistic regression assessed associations with 15 physical conditions. Dose–response relationships were evaluated based on the number of co‐occurring mental health conditions.

**Results:**

Four distinct profiles were identified: developmental delay/intellectual disability with mixed problems (DD/ID with MP, 8.0%), emotional problems (8.2%), attention‐deficit/hyperactivity disorder with behavioural problems (ADHD‐BP, 8.0%) and low mental health concerns (75.8%). The three clinical profiles exhibited a significant physical disease burden. DD/ID with MP showed the strongest associations with neurological and hearing conditions. Although emotional problems and ADHD‐BP shared a similar risk profile, emotional problems showed stronger associations with headaches and respiratory issues. A dose–response relationship was observed, with physical disease risk increasing for each additional mental health problem, particularly among children with > 3 disorders.

**Conclusions:**

Mental health multimorbidity is significantly associated with increased physical condition risk in a profile‐specific and dose‐dependent manner. This underscores the need for integrated clinical strategies that account for transdiagnostic patterns and comorbidity burden to enhance risk stratification and tailor interventions in paediatric populations.

## Abstract (highlights)

**WHAT IS ALREADY KNOWN ON THIS TOPIC**

- Mental health conditions are common in children and adolescents, and multimorbidity is frequent.
- Children with mental health problems are at increased risk of a range of physical health conditions.

**WHAT THIS STUDY ADDS**

- Four distinct profiles of mental health multimorbidity were identified, three of which exhibited different patterns of associations with physical health conditions. A clear dose–response relationship was found, with the risk of physical health conditions increasing with the number of co‐occurring mental health conditions, rising sharply beyond three conditions.

**HOW THIS STUDY MIGHT AFFECT RESEARCH, PRACTICE OR POLICY**

- These findings underscore the need to move beyond single‐diagnosis approaches and adopt integrated clinical practice and service‐delivery models that assess and manage both mental and physical health in paediatric populations.

## INTRODUCTION

The rising prevalence of mental health problems among children and adolescents poses a critical public health challenge globally, with profound implications for lifelong health trajectories.^1^, ^2^, ^3^, ^4^ Substantial evidence shows that mental health conditions are associated with an elevated risk of physical comorbidities in paediatric populations. However, existing research has predominantly focused on pairwise associations rather than complex patterns of multimorbidity.^5^, ^6^ Clinically, mental health comorbidities are highly prevalent, exemplified by the anxiety‐depression dyad in adolescents^7^ and the frequent co‐occurrence of attention‐deficit/hyperactivity disorder (ADHD) and conduct disorder in school‐aged children.^8^ These transdiagnostic patterns exhibit distinct pathophysiological signatures, which may be linked to physical health outcomes through shared neurobiological mechanisms, such as inflammatory pathway activation and dysregulation of the hypothalamic–pituitary–adrenal axis.^9^, ^10^, ^11^, ^12^ Understanding how these patterns of mental health multimorbidity are associated with physical health is crucial for advancing precision medicine in paediatric care, particularly given the potential for synergistic effects. Another important yet understudied factor is the cumulative burden of mental health multimorbidity, which may be associated with physical health through a dose–response relationship. Evidence suggests that a greater number of mental health diagnoses is associated with a higher overall physiological burden, which correlates with an elevated risk of co‐occurring physical health conditions through combined behavioural and biological mechanisms.^13^, ^14^ The number of co‐occurring mental health problems could serve as a practical measure for risk stratification in clinical settings, particularly for children and adolescents with multiple conditions requiring integrated care. However, its application in paediatric populations remains understudied, limiting the evidence‐based allocation of preventive resources.

Furthermore, children and adolescents aged 6–17 years represent a critical population for investigating the interactions between mental and physical health due to convergent epidemiological and developmental factors. During this period, children and adolescents undergo complex developmental transitions that intensify emotional and behavioural challenges, with the prevalence of mental health conditions rising markedly from elementary school through high school.^15^ As the brain matures, mental health symptoms tend to stabilise, making it easier to reliably identify co‐occurring conditions.^15^ At the same time, many chronic physical conditions first emerge during this period, shaping long‐term health and underscoring the importance of early identification.^16^ This convergence of growing psychiatric complexity, rising diagnostic stability and accelerating physical morbidity creates a critical intervention window. Early intervention with integrated care during this period can help identify modifiable risks and implement preventive strategies that lead to lifelong benefits.

This cross‐sectional study uses the nationally representative US National Survey of Children's Health (NSCH) to characterise the profiles of mental health multimorbidity and quantify their associations with physical conditions, while exploring dose–response relationships between mental health multimorbidity burden and physical health risks. The findings will inform the development of clinical protocols, prevention strategies and integrated care approaches for children and adolescents with complex conditions.

## METHODS

### Study design and data source

This cross‐sectional study analysed data from the 2023 NSCH, a nationally representative survey conducted by the US Census Bureau. The NSCH uses a complex, multistage sampling design to generate weighted estimates that accurately reflect the noninstitutionalised paediatric population aged 0–17 years across all 50 states and the District of Columbia. Data were systematically gathered through web‐ and mail‐based questionnaires, which were administered to parents or guardians, allowing for a comprehensive assessment of health metrics and sociodemographic factors in a population‐based context. As this research entailed a secondary analysis of de‐identified, publicly accessible data without direct participant interaction or primary data collection, ethical approval was not required, aligning with standard protocols for such epidemiological studies. The NSCH dataset was obtained through a formal request to the Child and Adolescent Health Measurement Initiative Data Resource Center, available at https://www.childhealthdata.org/dataset/download?rq=19119.

### Participants

**FIGURE 1:**
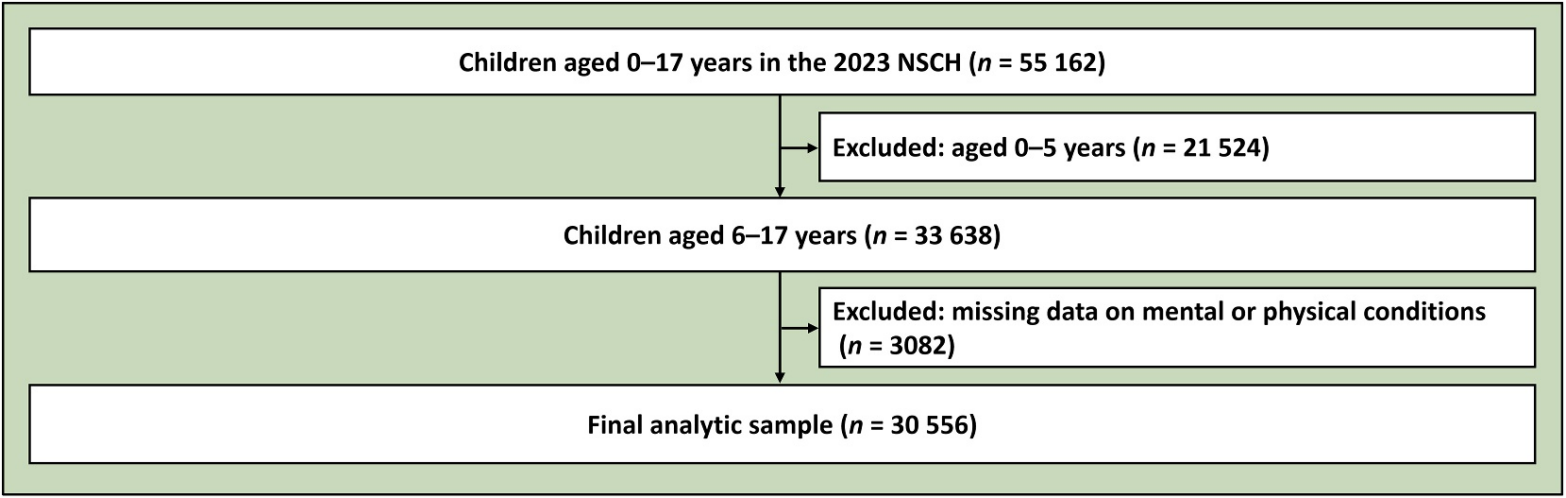
Flowchart of study participant selection from the 2023 NSCH. The diagram illustrates the derivation of the final analytic sample of children aged 6–17 years from the initial 2023 NSCH cohort, showing the number of participants at each stage of exclusion due to missing data on key variables. NSCH, National Survey of Children's Health.

Based on the 2023 NSCH, a total of 55 162 children aged 0–17 years were initially included in the survey. Given the higher prevalence and greater diagnostic stability of mental disorders in school‐aged children and adolescents,^15^ the study focused on the 6–17‐year age range, yielding a preliminary sample of 33 638 children. After excluding 3082 participants with missing data on mental or physical condition variables, the final analytic sample comprised 30 556 children aged 6–17 years. The flowchart of study participant selection is illustrated in figure 1.

### Measures

#### Mental health problems

The assessment of mental health problems used parent‐reported diagnostic history. Nine distinct diagnoses of mental health problems were evaluated based on lifetime diagnosis by a qualified healthcare provider: ADHD, autism spectrum disorder (ASD), developmental delay/intellectual disability (DD/ID), Tourette syndrome, speech disorders, learning disabilities, behavioural problems (BPs), depression and anxiety. These conditions represent the most commonly diagnosed disorders identified in paediatric psychiatric research. Diagnoses were ascertained through caregiver affirmation that a healthcare provider had diagnosed the child with each condition, yielding binary (yes/no) variables for analysis. The use of parental reports of professionally assigned diagnoses is a widely accepted approach in large‐scale epidemiological studies of child psychopathology.^17^

#### Physical conditions

The inclusion of these conditions, all based on parent‐reported provider diagnoses, was contingent on the specific condition, with criteria encompassing either a diagnosis within the past 12 months or a lifetime history. Fifteen physical health conditions were evaluated, encompassing a range of chronic and acute health issues prevalent in paediatric populations. The specific conditions included neurological (cerebral palsy, epilepsy/seizure disorders, headaches and concussions/brain injuries), heart condition, respiratory (breathing or other respiratory problems), gastrointestinal (digestive disorders and eating/swallowing difficulties), blood, immune (autoimmune diseases, allergies and asthma) and sensory (blindness/visual impairment, deafness/hearing impairment and chronic pain).

#### Covariates

We included several key covariates to account for potential confounding factors known to influence both mental and physical health outcomes in paediatric populations. Child‐level characteristics consisted of age (modelled as a continuous variable in years), sex (boy or girl) and preterm status (defined as gestational age < 37 weeks, dichotomised as yes/no). Race and ethnicity were categorised into the following five groups: Hispanic (any race), non‐Hispanic White, non‐Hispanic Black, non‐Hispanic Asian and non‐Hispanic other or multiracial. Family‐level covariates encompassed the highest education level in the household, classified as high school graduation or less versus any education beyond high school; marital status of the responding parent, operationalised as living with a partner or living alone; and annual family income relative to the federal poverty level (FPL), grouped into four tiers: 0%–99%, 100%–199%, 200%–399% and ≥ 400% of the FPL. Multiple imputation was applied for covariates with missing values.

### Statistical analysis

Statistical analyses were conducted to examine the associations between mental health multimorbidity and physical conditions in children. Latent class analysis (LCA) was employed to identify distinct subgroups based on nine mental health conditions: ADHD, ASD, DD/ID, depression, anxiety, Tourette syndrome, BPs, speech disorders and learning disabilities. Model fit was assessed using the Akaike information criterion (AIC), Bayesian information criterion (BIC), entropy and the size of the smallest class. The four‐class model was selected as optimal, balancing statistical fit and clinical interpretability (Supporting Information S1: table S1). The resulting profiles were labelled as ‘Low Mental Health Concerns’, ‘DD/ID with Mixed Problems’, ‘Emotional Problems’ and ‘ADHD with Behavioural Problems’. To validate the robustness and clinical coherence of the LCA‐derived profiles, hierarchical cluster analysis (HCA) was performed using the nine binary mental health indicators, with squared Euclidean distance and Ward's minimum variance method. HCA is appropriate for binary comorbidity data and yields an independent, data‐driven, clinically interpretable grouping structure.

Multivariable logistic regression was used to assess associations between latent class membership (a four‐category exposure variable) and physical conditions, with all four profiles included in a single model and the ‘Low Mental Health Concerns’ group as the reference. Results were expressed as adjusted odds ratios (aORs) with 95% confidence intervals (CIs). All models were adjusted for child characteristics (age, sex, gestational age, race and ethnicity) and family characteristics (household education, marital status and family income level). A dose–response analysis was performed using ordinal logistic regression to evaluate the relationship between the number of co‐occurring mental health conditions and physical conditions, treating the number as an ordinal variable with five levels (0, 1, 2, 3 and > 3) and using the group with zero mental health conditions as the reference, while adjusting for the same covariates.

To test the robustness of the findings, sensitivity analyses were conducted. First, stratified analyses by sex were performed to examine potential effect modification. Second, given baseline characteristic differences between children with and without missing data on mental health problems and physical conditions (Supporting Information S1: table S2), we repeated both LCA and regression models using multiple imputation in the full sample.

All analyses accounted for the complex survey design of the NSCH, incorporating the provided survey weights, strata and primary sampling units to ensure nationally representative estimates. All analyses were performed using R version 4.4.0. The poLCA package was used for LCA, and the mice package was used for multiple imputation. Regression modelling was carried out using standard functions within the R stats package. A two‐sided significance level of 5% (*p* < 0.05) was used for all statistical tests.

## RESULTS

### Burden of mental health multimorbidity in US children aged 6–17 years

**FIGURE 2:**
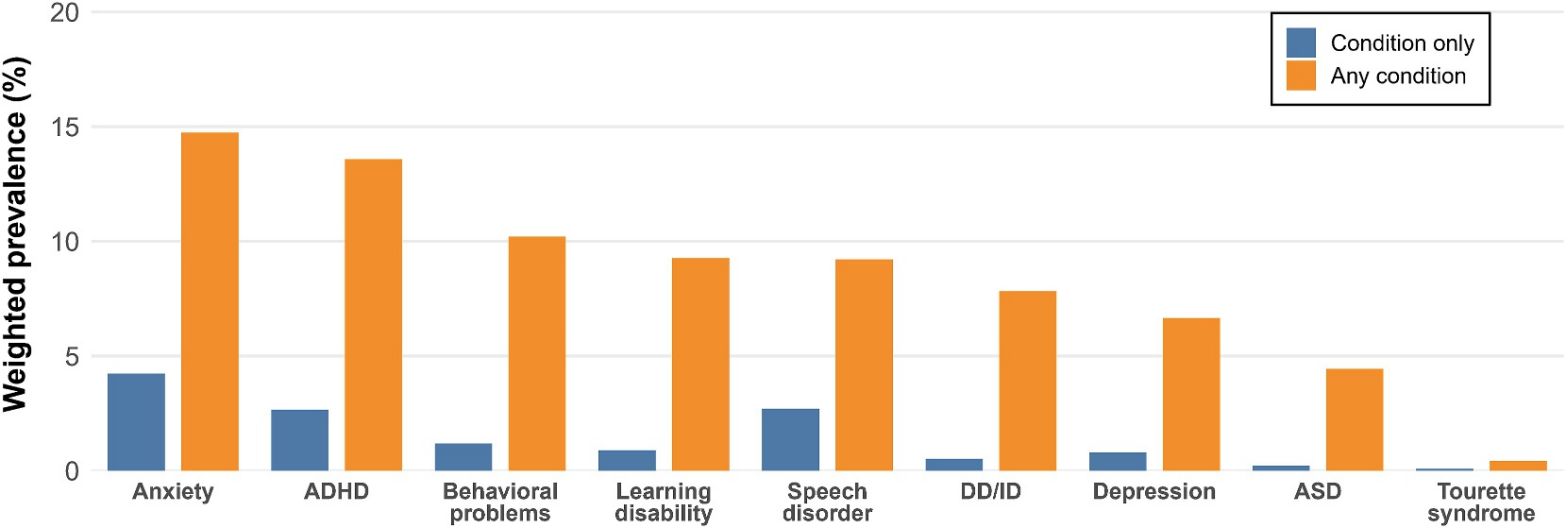
Weighted prevalence of mental health conditions in US children aged 6–17 years. Estimates are weighted to be nationally representative. ‘Condition only’ (blue) indicates children who have the specified condition and no other comorbid mental health conditions among the nine evaluated in this study. ‘Any condition’ (orange) indicates children with the specified condition, regardless of whether they also have other comorbidities. ADHD, attention‐deficit/hyperactivity disorder; ASD, autism spectrum disorder; DD/ID, developmental delay/intellectual disability.

The 2023 NSCH data showed a substantial burden of co‐occurring mental health problems among US children aged 6–17 years. As shown in figure 2 and detailed in Supporting Information S1: table S3, the prevalence of co‐occurring mental health conditions significantly exceeded that of isolated conditions across all diagnostic categories. For example, although the total weighted prevalence of anxiety disorders was 14.7%, only 4.2% of children had anxiety as an isolated condition. Similarly, ADHD showed a total weighted prevalence of 13.6%, with isolated ADHD present in just 2.7% of children. This pattern was consistent for BPs (total 10.2%, isolated 1.2%), learning disabilities (total 9.3%, isolated 0.9%), speech disorders (total 9.2%, isolated 2.7%), depression (total 6.7%, isolated 0.8%), DD/ID (total 7.8%, isolated 0.5%), ASD (total 4.4%, isolated 0.2%) and Tourette syndrome (total 0.4%, isolated 0.1%), indicating that co‐occurrence represents the predominant clinical presentation for common mental health conditions.

The specific patterns of pairwise co‐occurrence are illustrated in Supporting Information S1: figure S1 and table S4. Among dyadic combinations, ADHD frequently co‐occurred with anxiety disorders (5.8%), BPs (6.5%) and learning disabilities (5.2%). Anxiety disorders commonly co‐occurred with depression (5.2%). DD/ID demonstrated frequent co‐occurrence with learning disabilities (5.1%) and speech disorders (4.4%). These data reveal distinct patterns of mental health multimorbidity, with certain diagnostic pairs occurring more frequently than others.

### Profiles of mental health multimorbidity in US children aged 6–17 years

**FIGURE 3:**
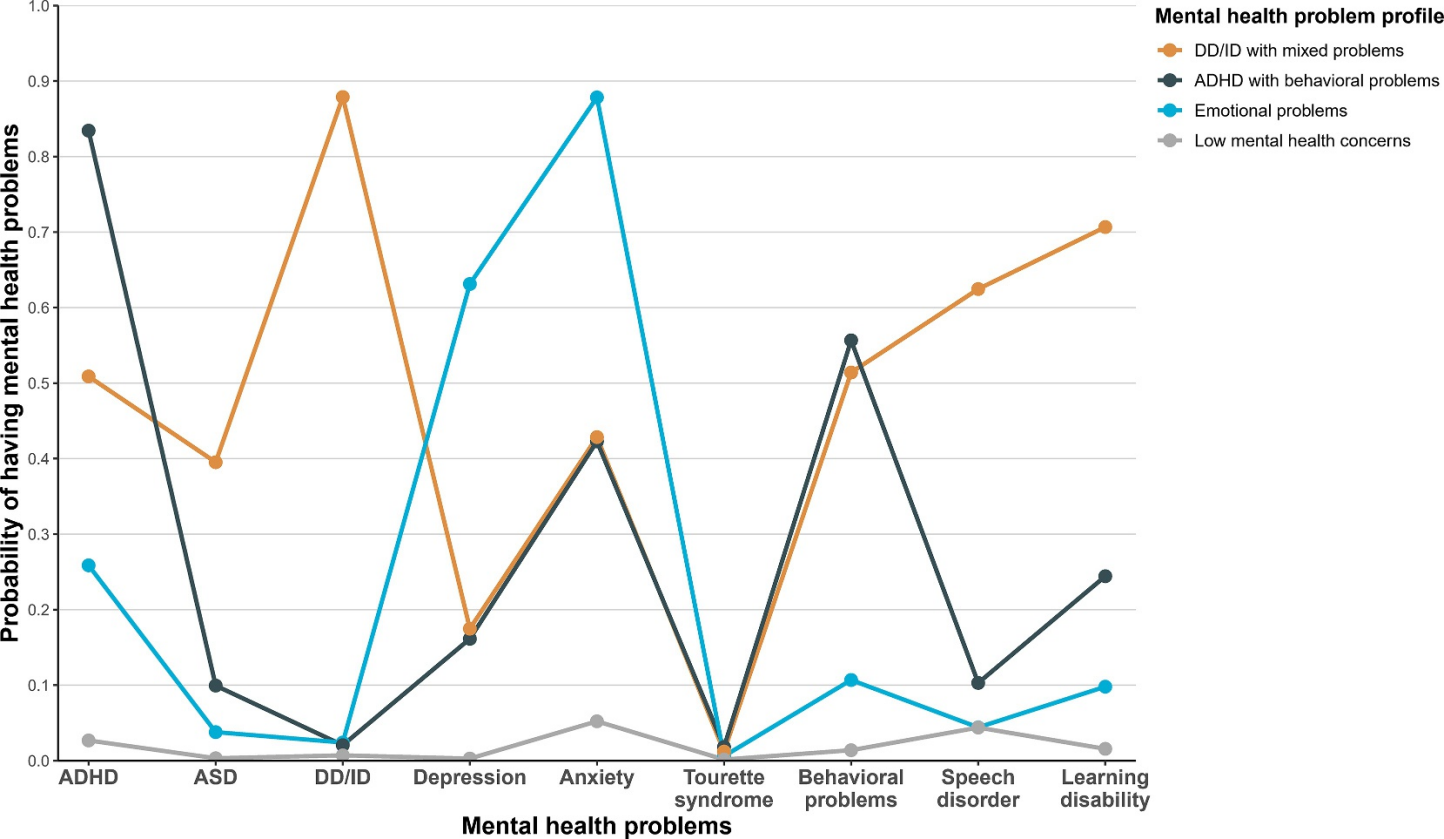
Profiles of mental health multimorbidity identified through latent class analysis in US children aged 6–17 years. ADHD, attention‐deficit/hyperactivity disorder; ASD, autism spectrum disorder; DD/ID, developmental delay/intellectual disability.

LCA of mental health problems identified four distinct profiles of multimorbidity among US children aged 6–17 years within the nationally representative NSCH dataset. Model fit statistics supported the 4‐class solution (Supporting Information S1: table S1). Among all candidate models, the 4‐class model achieved a favourable balance of statistical fit, classification precision and practical utility. It maintained relatively low AIC/BIC values, high entropy (0.874), indicative of clear classification, and an adequately sized smallest class (8.0%) for robust subgroup characterisation, while aligning well with clinical interpretability. Figure 3 details the conditional probabilities defining each profile (sample size and prevalence shown below and detailed in table 1): (1) DD/ID with mixed problems (MPs) (*n* = 2435, 8.0%), characterised by high probabilities of DD/ID, speech disorders and learning disabilities, along with moderate probabilities of ADHD, ASD, anxiety and BPs; (2) emotional problems (*n* = 2511, 8.2%), marked predominantly by high probabilities of anxiety and depression; (3) ADHD with BPs (*n* = 2438, 8.0%), defined by high probabilities of ADHD, behavioural problems and moderate anxiety; and (4) low mental health concerns (*n* = 23 172, 75.8%), with low probabilities across all assessed conditions. HCA was performed to validate the identified multimorbidity profiles. HCA identified a four‐cluster structure highly consistent with the LCA solution (Supporting Information S1: figure S2; table S5). HCA Cluster 1 corresponded to the low mental health concerns profile; Cluster 2 aligned with the ADHD with BPs profile; Cluster 3 matched the DD/ID with MPs profile; and Cluster 4 represented the emotional problems profile. These results confirm the robustness and clinical coherence of the LCA‐derived multimorbidity profiles. In the sensitivity analysis, these profiles of mental health multimorbidity remained consistent when all individuals in the full sample were included, regardless of missing data on mental health or physical condition variables (Supporting Information S1: figure S3).

**TABLE 1:** Characteristics of children by four profiles of mental health problems

	Low mental health concerns (*N* = 23 172)	DD/ID with mixed problems (*N* = 2435)	Emotional problems (*N* = 2511)	ADHD with behavioural problems (*N* = 2438)	Statistic	*p* value
Age (years), mean (SD)	11.6 (3.52)	11.3 (3.48)	15.1 (1.94)	11.8 (3.25)	*F* = 846.40	< 0.001
Sex, *n* (%)						
Boy	11 860 (51.2)	1673 (68.7)	453 (18.0)	1896 (77.8)	*χ* ^2^ = 2087.20	< 0.001
Girl	11 312 (48.8)	762 (31.3)	2058 (82.0)	542 (22.2)		
Preterm, *n* (%)	2160 (9.3)	475 (19.5)	278 (11.1)	296 (12.1)	*χ* ^2^ = 252.27	< 0.001
Race and ethnicity, *n* (%)					*χ* ^2^ = 365.92	< 0.001
Hispanic/Latino	3535 (15.3)	372 (15.3)	341 (13.6)	314 (12.9)		
White, non‐Hispanic	14 621 (63.1)	1498 (61.5)	1821 (72.5)	1789 (73.4)		
Black, non‐Hispanic	1447 (6.2)	213 (8.7)	115 (4.6)	125 (5.1)		
Asian, non‐Hispanic	1837 (7.9)	120 (4.9)	56 (2.2)	38 (1.6)		
Other/multiracial, non‐Hispanic	1732 (7.5)	232 (9.5)	178 (7.1)	172 (7.1)		
Education in household, *n* (%)					*χ* ^2^ = 47.54	< 0.001
High school or less	3344 (14.4)	455 (18.7)	325 (12.9)	304 (12.5)		
More than high school	19 828 (85.6)	1980 (81.3)	2186 (87.1)	2134 (87.5)		
Marital status, *n* (%)					*χ* ^2^ = 421.07	< 0.001
Living with a partner	19 401 (83.7)	1805 (74.1)	1817 (72.4)	1771 (72.6)		
Living alone	3771 (16.3)	630 (25.9)	694 (27.6)	667 (27.4)		
Family income relative to FPL, *n* (%)					*χ* ^2^ = 280.73	< 0.001
0%–99% of FPL	2726 (11.8)	476 (19.5)	291 (11.6)	316 (13.0)		
100%–199% of FPL	3590 (15.5)	524 (21.5)	425 (16.9)	426 (17.5)		
200%–399% of FPL	6850 (29.6)	729 (29.9)	794 (31.6)	756 (31.0)		
≥ 400% of FPL	10 006 (43.2)	706 (29.0)	1001 (39.9)	940 (38.6)		

*Note*: The continuous variable (age) was compared using one‐way analysis of variance (ANOVA) (reported as the *F*‐statistic), and categorical variables were compared using Pearson's chi‐square test (reported as the *χ* ^2^‐statistic). All tests were two‐sided, with a significance level of *p* < 0.05.

Abbreviations: ADHD, attention‐deficit/hyperactivity disorder; DD/ID, developmental delay/intellectual disability; FPL, federal poverty level; SD, standard deviation.

Baseline characteristics of the four profiles of mental health multimorbidity are shown in table 1. The low mental health concerns profile, representing the largest subgroup, served as the reference group in subsequent analyses. All three clinical profiles showed higher proportions of preterm birth and single‐parent households compared with the reference group. Children in the DD/ID with MPs profile were more likely to be boys (68.7% vs. 51.2%), to have been born preterm (19.5% vs. 9.3%), to have parents with a low education level (18.7% vs. 14.4%), to live in a single‐parent household (25.9% vs. 16.3%) and to live in a low‐income family below 200% of the FPL (41.0% vs. 27.3%). Participants in the emotional problems profile were predominantly girls (82.0% vs. 48.8%), were more likely to be adolescents (mean age 15.1 vs. 11.6 years) and had a higher proportion of non‐Hispanic White children (72.5% vs. 63.1%). Children in the ADHD with BPs profile had a higher proportion of boys (77.8% vs. 51.2%) and were more likely to be non‐Hispanic White (73.4% vs. 63.1%).

### Associations of mental health multimorbidity profiles with physical conditions in US children aged 6–17 years

As illustrated in Supporting Information S1: figure S4, systematic evaluation revealed broad and significant associations between various mental health problems and a wide range of physical conditions. With the exception of Tourette syndrome, all other mental health problems, including DD/ID, ADHD, ASD, depression, anxiety, BPs, speech disorders and learning disabilities, were associated with an increased burden of somatic conditions. These findings emphasise the pervasive nature of physical health challenges among children with mental health problems.

**FIGURE 4:**
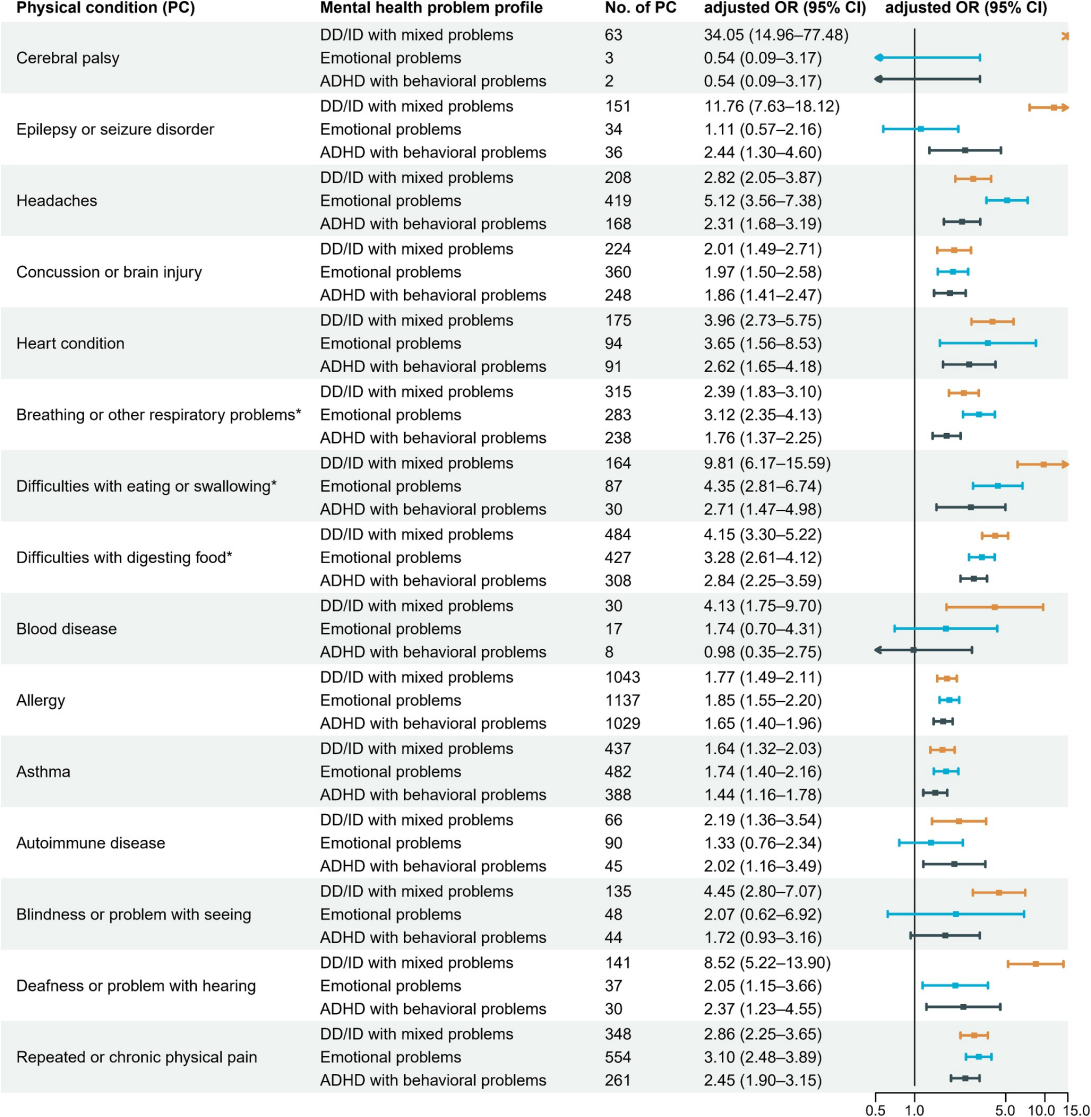
Associations of profiles of mental health multimorbidity with physical conditions in US children aged 6–17 years (weighted for complex survey design). Results were based on joint modelling of latent class analysis of mental health problems and logistic regression of physical conditions on class membership. Logistic regression models were weighted to account for the complex survey design of the NSCH, including strata, clusters and sampling weights, and adjusted for children's characteristics (age, sex, gestational age, race and ethnicity) and family characteristics (education level, marital status and family income). *Diagnosed within the past 12 months. ADHD, attention‐deficit/hyperactivity disorder; CI, confidence interval; DD/ID, developmental delay/intellectual disability; NSCH, National Survey of Children's Health; OR, odds ratio.

As detailed in figure 4, the DD/ID with MPs profile demonstrated particularly strong associations with numerous physical conditions, including cerebral palsy (aOR = 34.05; 95% CI 14.96–77.48), epilepsy or seizure disorder (aOR = 11.76; 95% CI 7.63–18.12), heart condition (aOR = 3.96; 95% CI 2.73–5.75), difficulties with eating or swallowing (aOR = 9.81; 95% CI 6.17–15.59), difficulties with digesting food (aOR = 4.15; 95% CI 3.30–5.22), blood disease (aOR = 4.13; 95% CI 1.75–9.70), blindness or problems with seeing (aOR = 4.45; 95% CI 2.80–7.07) and deafness or problems with hearing (aOR = 8.52; 95% CI 5.22–13.90). Particularly, the strength of the associations with neurological conditions (e.g., cerebral palsy and epilepsy or seizure disorder) and deafness or problems with hearing was significantly greater in the DD/ID with MPs profile than in either the emotional problems or ADHD with BPs profiles. Although both the emotional problems and ADHD with BPs profiles were associated with a similar spectrum of physical conditions, the emotional problems profile showed stronger links to several somatic domains relative to the ADHD with BPs profile. These included headaches (aOR = 5.12; 95% CI 3.56–7.38 vs. aOR = 2.31; 95% CI 1.68–3.19) and breathing or other respiratory problems (aOR = 3.12; 95% CI 2.35–4.13 vs. aOR = 1.76; 95% CI 1.37–2.25). These results highlight a profile‐specific pattern of somatic comorbidity among children with different mental health conditions. Sensitivity analyses confirmed that these association patterns remained similar across sex‐stratified subgroups (Supporting Information S1: figures S5 and S6) and in the full sample, including individuals with missing data on mental or physical health variables (Supporting Information S1: figure S7).

### Dose–response relationship between mental health multimorbidity and physical conditions in US children aged 6–17 years

**FIGURE 5:**
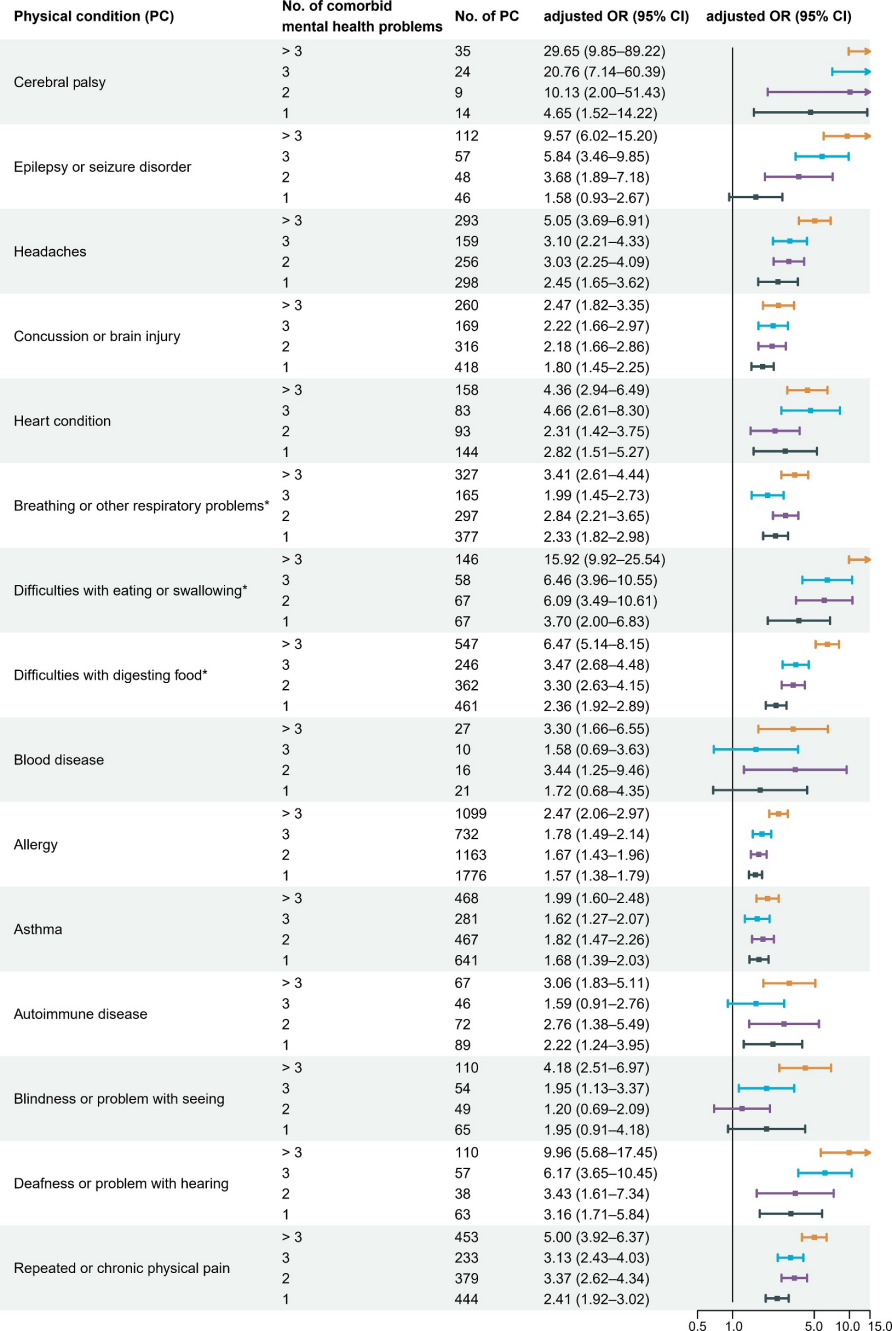
Associations between the number of mental health multimorbidities and physical conditions in US children aged 6–17 years (weighted for complex survey design). Logistic regression models were weighted to account for the complex survey design of the NSCH, including strata, clusters and sampling weights, and adjusted for children's characteristics (age, sex, gestational age, race and ethnicity) and family characteristics (education level, marital status and family income). *Diagnosed within the past 12 months. CI, confidence interval; NSCH, National Survey of Children's Health; OR, odds ratio.

A clear dose–response relationship was observed between the number of co‐occurring mental health conditions and the risk of physical conditions. As detailed in figure 5, increasing mental health multimorbidity was consistently associated with elevated odds of physical conditions across all domains examined. This graded escalation in risk was most pronounced when more than three mental health conditions were present, underscoring the cumulative impact of mental health multimorbidity on physical health. Notably, the increase in risk was particularly striking for neurological, digestive and sensory conditions. For cerebral palsy, the aOR demonstrated substantial increases: 10.13 (95% CI 2.00–51.43) for two mental health conditions, 20.76 (95% CI 7.14–60.39) for three conditions and 29.65 (95% CI 9.85–89.22) for more than three conditions. Similarly, for epilepsy or seizure disorder, the aOR rose from 3.68 (95% CI 1.89–7.18) for two conditions to 5.84 (95% CI 3.46–9.85) for three conditions, reaching 9.57 (95% CI 6.02–15.20) for more than three conditions. Regarding digestive conditions, for difficulties with eating or swallowing, the aOR rose from 6.09 (95% CI 3.49–10.61) for two conditions to 6.46 (95% CI 3.96–10.55) for three conditions, reaching 15.92 (95% CI 9.92–25.54) for more than three conditions; for difficulties with digesting food, the aOR rose from 3.30 (95% CI 2.63–4.15) for two conditions to 3.47 (95% CI 2.68–4.48) for three conditions, reaching 6.47 (95% CI 5.14–8.15) for more than three conditions. Parallel patterns emerged for sensory impairments: the adjusted odds ratios (aORs) for blindness or problems with seeing increased from 1.20 (95% CI 0.69–2.09) for two conditions to 1.95 (95% CI 1.13–3.37) for three conditions and 4.18 (95% CI 2.51–6.97) for more than three conditions. Similarly, the aORs for deafness or problems with hearing escalated from 3.43 (95% CI 1.61–7.34) for two mental health conditions to 6.17 (95% CI 3.65–10.45) for three conditions and 9.96 (95% CI 5.68–17.45) for more than three conditions. These results highlight a threshold effect at > 3 disorders, where physical health risks, especially for severe neurological, digestive and sensory conditions, became markedly elevated relative to lower degrees of multimorbidity. This dose–response pattern was maintained in sex‐stratified analyses (Supporting Information S1: figures S8 and S9) and in the full sample, incorporating individuals with incomplete data on mental or physical health measures (Supporting Information S1: figure S10).

## DISCUSSION

### Main findings

This national study identified four distinct profiles of mental health multimorbidity among US children aged 6–17 years: (1) DD/ID with MPs, (2) ADHD with BPs, (3) emotional problems and (4) low mental health concerns. Three of these profiles were associated with a significant burden of physical conditions. The DD/ID with MPs profile showed the strongest associations, particularly with neurological and hearing conditions, with these risks significantly greater than those observed in the other profiles. The emotional problems and ADHD with BPs profiles were associated with a similar spectrum of physical conditions; however, the emotional problems profile showed stronger links to headaches and respiratory issues. Additionally, we found a dose–response relationship between mental health multimorbidity and physical health risk, with the greatest escalation in risk occurring in individuals with more than three co‐occurring mental health conditions.

The identification of these four distinct profiles through LCA represents a significant methodological advancement beyond traditional comorbidity studies, which typically focus on dyadic relationships between specific disorders. By examining patterns of co‐occurrence across multiple mental health conditions simultaneously, we revealed clinically meaningful subgroups that better reflect the real‐world complexity of paediatric mental health presentations, an insight that discrete diagnostic categories alone cannot capture.

This study identifies the DD/ID with MPs profile as a clinically significant subgroup, representing nearly 8% of children in this nationally representative sample. This profile is characterised by the combined and integrated co‐occurrence of multiple neurodevelopmental disorders (e.g., DD/ID, speech disorders, learning disabilities, ADHD and ASD), alongside anxiety and BPs. This pattern reflects a combined clinical presentation rather than a condition‐specific one, thereby reinforcing its consistency with the recognised comorbidity landscape within the DD/ID population.^18^, ^19^, ^20^, ^21^, ^22^ Moreover, although prior research has often examined isolated mental health comorbidities, our phenomapping approach provides an integrated characterisation of this complex cluster and underscores the frequently overlooked role of anxiety within it.

Regarding somatic correlates, the DD/ID with MPs profile demonstrated particularly strong associations with neurological conditions. These findings align with existing literature that reports elevated rates of neurological manifestations, motor deficits and structural brain abnormalities among individuals with complex neurodevelopmental presentations, including co‐occurring ASD and related conditions.^23^, ^24^ Furthermore, although each of the nine individual mental health problems examined in this study was associated with sensory deficits, the risks of hearing conditions were markedly elevated within the DD/ID with MPs profile compared with those in the other profiles, a pattern that has not been previously highlighted. Although hearing abnormalities have been recognised as features of certain genetic DD/ID syndromes,^25^, ^26^ the current results indicate that the observed somatic associations are more closely linked to the overall configuration of mental health multimorbidity than to DD/ID alone.

In addition, we identified a distinct profile termed ADHD with BPs, characterised by co‐occurring behavioural disturbances and prominent anxiety symptoms. This empirically derived subgroup underscores a clinically prevalent ADHD presentation involving both behavioural and emotional dysregulation. The findings align with prior research indicating that complex ADHD phenotypes often involve interactions between externalising (e.g., behavioural and conduct disorders) and internalising (e.g., anxiety) symptoms,^8^ and contribute to a deeper understanding of the heterogeneous clinical manifestations of ADHD.

In terms of physical health, this profile demonstrated broad associations with multiple somatic conditions, including neurological, heart, sensory, respiratory, gastrointestinal, pain‐related and immunological conditions. These findings are consistent with previous reports^27^, ^28^ and extend the evidence linking ADHD to multisystem physiological dysregulation. Although prior studies have primarily emphasised visual and auditory attentional deficits in ADHD, typically manifesting as distractibility and sensory hypersensitivity, emerging evidence points to a broader spectrum of sensory processing abnormalities, including visual impairment, auditory dysfunction and elevated pain sensitivity.^29^, ^30^, ^31^ Our results support this expanded understanding of sensory dysfunction in ADHD, highlighting its clinical relevance due to its pervasive impacts on daily functioning, including associations with emotional dysregulation and functional impairments in school and family settings.^29^, ^32^ Further investigation into the underlying neurophysiological and sensory integration mechanisms may elucidate ADHD pathophysiology and inform targeted interventions.

Prior research has linked childhood emotional symptoms to specific issues such as asthma and dermatitis.^6^, ^33^, ^34^ This study extends the evidence by showing that the emotional problems profile, primarily characterised by anxiety and depression and more prevalent in adolescents, is strongly associated with a wider range of physical health outcomes. Although the emotional problems profile and the ADHD with BPs profile were associated with a similar spectrum of physical conditions, the emotional problems profile consistently showed stronger associations with headaches and respiratory issues. This profile‐specific association pattern implies that the connections between emotional and physical symptoms may reflect distinct underlying aetiological mechanisms. Potential mechanisms may include stress‐related physiological processes that amplify pain sensitivity and visceral distress, likely influenced by shared genetic and environmental risk factors.^12^, ^35^, ^36^, ^37^ These results underscore the importance of bidirectional screening between adolescent anxiety/depression and specific physical conditions, enabling more targeted interventions.

A clear dose–response relationship was observed between the number of co‐occurring mental health conditions and physical condition risk, with a pronounced threshold at > 3 co‐occurring conditions. Risk increased gradually with 1–3 conditions but rose significantly beyond this point, especially for neurological, digestive and sensory conditions. This pattern highlights that children with more than three mental health problems represent a high‐priority group that needs enhanced physical health screening and integrated mental–physical health management in clinical settings.

Our findings suggest that mental health multimorbidity profiles, particularly the DD/ID with MPs and ADHD with BPs, are primarily neurodevelopmental in origin. Their associations with physical conditions may reflect shared genetic and developmental mechanisms.^23^, ^38^, ^39^ Evidence indicates that neurodevelopmental and physical disorders share common genetic underpinnings, leading to pleiotropic effects, whereby genetic factors simultaneously influence both mental and somatic health.^40^, ^41^, ^42^ Moreover, the relationship between mental and physical health is bidirectional, involving complex pathways such as dysregulated immune and inflammatory processes, neuroendocrine dysfunction and gut–brain axis disruptions.^43^, ^44^, ^45^ This interplay indicates that comorbid conditions may share overlapping genetic and pathophysiological origins, highlighting the need for integrated clinical strategies to address the multifaceted health burdens in paediatric populations.

This study has several strengths, including the use of a large, nationally representative sample, which enhances the generalisability of our findings to the broader US paediatric population. The application of LCA offers a data‐driven, person‐centred approach to identify clinically meaningful mental health multimorbidity profiles that capture real‐world heterogeneity beyond traditional diagnostic categories.

### Limitations

However, several limitations must be acknowledged. The cross‐sectional design precludes causal inference regarding the direction of these relationships between mental and physical health conditions; future longitudinal studies are needed to establish temporal precedence and clarify bidirectional pathways. Furthermore, we acknowledge that the observed associations between mental health multimorbidity and physical conditions may be attributable to three key noncausal factors: the presence of underlying neurodevelopmental syndromes that confer concurrent risk for both mental and physical morbidity^39^; shared social determinants (e.g., low socioeconomic status and limited healthcare access) that are associated with elevated risk across both mental and physical health domains^46^; and potential surveillance bias, whereby children with diagnosed mental health conditions may receive more frequent clinical monitoring, leading to higher detection rates of physical conditions compared with children without mental health concerns. Although parent‐reported diagnoses in large‐scale surveys can be subject to measurement error, the NSCH's use of a standardised data collection instrument helps to mitigate this issue; future investigations integrating parental reports with electronic health record data containing clinician‐assigned diagnostic codes and objective clinical assessments would further strengthen the reliability of paediatric health condition measurement and address residual biases associated with caregiver‐reported data. Residual confounding may persist due to unmeasured genetic, environmental or biological factors, although we adjusted for a wide range of covariates. We additionally note that ID is categorised as a single binary variable in the NSCH dataset with no stratification by clinical severity, and the dataset lacks validated assessments of functional impairment for this condition. Subsequent research incorporating granular ID severity data and validated functional status measures would better characterise the heterogeneity of this condition and its differential associations with physical health outcomes. Besides, although the sample was large, statistical power remained limited for examining very rare physical conditions. Future studies with targeted recruitment or linked health records could address lower‐prevalence outcomes. Finally, the generalisability of these findings to other countries requires careful consideration. Direct applicability may be limited by cross‐national differences in healthcare systems, diagnostic practices, cultural perceptions of mental health and socioeconomic contexts. Nevertheless, our core finding that children with complex, multidomain mental health problems represent a distinct high‐risk subgroup for severe physical comorbidities carries clear public health significance. This pattern highlights a shared paediatric health challenge. Further research across diverse national settings is needed to validate these associations and to inform globally responsive public health strategies.

### Implications

In conclusion, this study identifies four distinct profiles of mental health multimorbidity among US children aged 6–17 years and highlights their differential associations with physical conditions, underscoring that transdiagnostic patterns are closely associated with physical health outcomes. The DD/ID with MPs profile showed the strongest associations with neurological and hearing conditions. Although the emotional problems profile and ADHD with BPs profile were associated with a similar range of physical conditions, the emotional problems profile exhibited stronger links to headaches and respiratory issues. A clear dose–response relationship was observed between the number of co‐occurring mental health conditions and the risk of physical conditions, with markedly elevated odds in the presence of more than three mental health conditions. This indicates that a higher multimorbidity burden is significantly associated with increased susceptibility to physical conditions. These findings emphasise the need for integrated clinical approaches that consider profile‐specific patterns and the overall comorbidity burden to address the complexities of mental and physical health in paediatric care. Future longitudinal research is warranted to clarify underlying mechanisms and causal pathways, ultimately guiding targeted early intervention and resource allocation for children with multimorbid conditions.

## AUTHOR CONTRIBUTIONS

All authors contributed significantly to this research. The statistical expert for this research: Hua He. Concept and design: Fei Li and Guodong Ding. Acquisition, analysis or interpretation of data: Hua He and Feng Li. Drafting of the manuscript: Hua He. Critical revision of the manuscript for important intellectual content: all authors. Supervision: Fei Li and Guodong Ding.

## FUNDING

This work was supported by the National Natural Science Foundation of China under Grant Nos. 82430104, 82125032, 81930095, 82204064 and 82001771; the China Brain Initiative under Grant No. 2021ZD0200800; the Science and Technology Commission of Shanghai Municipality under Grant Nos. 19410713500, 23Y21900500 and 2018SHZDZX01; the Shanghai Municipal Commission of Health and Family Planning under Grant Nos. GWVI‐11.1‐34, 2020CXJQ01 and 2018YJRC03; the Shanghai Municipal Health Commission under Grant Nos. 20214Y0125 and GWVI‐11.2‐YQ30; the Innovative Research Team of High‐Level Local Universities in Shanghai under Grant No. SHSMU‐ZDCX20211100; and the Shanghai Pujiang Program under Grant No. 22PJD045. The funding sources had no role in the design and conduct of the study; collection, management, analysis and interpretation of the data; preparation, review or approval of the manuscript; and decision to submit the manuscript for publication.

## CONFLICT OF INTEREST STATEMENT

The authors declare no conflicts of interest.

## ETHICS STATEMENT

This study utilised de‐identified, publicly available data from the 2023 National Survey of Children's Health (NSCH). The original NSCH data collection was approved by the U.S. Census Bureau's Institutional Review Board. The secondary analysis of these data was deemed exempt from review by the Institutional Review Board of Xinhua Hospital Affiliated to Shanghai Jiao Tong University School of Medicine, as it did not constitute human subjects research as defined by relevant ethical guidelines.

## Supporting information

Supporting Information S1

## Data Availability

Data supporting this study are derived from the publicly available National Survey of Children's Health (NSCH) dataset and can be accessed upon request through the official NSCH data repository (https://www.childhealthdata.org).
