# Supplementary material for: Mental health multimorbidity and physical conditions in children aged 6–17 years in the United States
Source: Gen Psychiatr. 2026 Aug 17;39(4):e70048. doi: 10.1002/gps3.70048 (PMC13481711; doi:10.1002/gps3.70048)
Supplement: Supplementary file 1 [file GPS3-39-e70048-s001.pdf]

Table S1. Model fit statistics for latent class analyses

	AIC	BIC	Classification probabilities	Size of smallest class	Entropy
2-class	138 636	138 845	0.93-0.96	19.5%	0.839
3-class	132 320	132 661	0.83-0.97	10.3%	0.860
4-class	129 844	130 319	0.82-0.96	8.0%	0.874
5-class	129 277	129 885	0.75-0.96	1.9%	0.872

AIC = Akaike Information Criterion; BIC = Bayesian Information Criterion.

Table S2. Characteristics of children by missing status for mental health problem or physical condition variables.

	No missing (N = 30 586)	missing (N = 3052)	Statistic	*p*- value
**Age (years), mean (SD)**	11.9 (3.53)	12.1 (3.52)	t = -3.92	< 0.001
**Sex, n (%)**			χ^2 = 2.41	0.121
Boy	15 899 (52.0%)	1541 (50.5%)		
Girl	14 687 (48.0%)	1511 (49.5%)		
**preterm, n (%)**	3215 (10.5%)	336 (11.0%)	χ^2 = 0.68	0.411
**Race and Ethnicity, n (%)**			χ^2 = 129.48	< 0.001
Hispanic/Latino	4568 (14.9%)	578 (18.9%)		
White,non-Hispanic	19 748 (64.6%)	1729 (56.7%)		
Black,non-Hispanic	1902 (6.2%)	305 (10.0%)		
Asian,non-Hispanic	2052 (6.7%)	246 (8.1%)		
Other/Multi-racial, non-Hispanic	2316 (7.6%)	194 (6.4%)		
**Education in household, n (%)**			χ^2 = 82.54	< 0.001
High school or less	4432 (14.5%)	631 (20.7%)		
More than high school	26 154 (85.5%)	2421 (79.3%)		
**Marital status, n (%)**			χ^2 = 24.91	< 0.001
living with a partner	24 818 (81.1%)	2362 (77.4%)		
Living alone	5768 (18.9%)	690 (22.6%)		
**Family income relative to FPL, n (%)**			χ^2 = 91.25	< 0.001
0–99% of FPL	3812 (12.5%)	534 (17.5%)		
100–199% of FPL	4969 (16.2%)	580 (19.0%)		
200–399% of FPL	9139 (29.9%)	822 (26.9%)		
≥ 400% of FPL	12 666 (41.4%)	1116 (36.6%)		

FPL = federal poverty level; SD = standard deviation.

Continuous variables (age) were compared using t-test (reported as t-statistic), and categorical variables were compared using Pearson's chi-square test (reported as χ^2-statistic). All tests were two-sided, with a significance level of $p < 0.05$.

Table S3. Numbers and weighted prevalence of mental health conditions: isolated vs. total cases in US children aged 6–17 years.

Mental health problems	Condition only			Any Condition		
	Unweighted No.	Weighted No.	Weighted prevalence (%) (95% CI)	Unweighted No.	Weighted No.	Weighted prevalence (%) (95% CI)
**Anxiety**	1624	1 905 220	4.2 (3.9, 4.6)	5742	6 640 755	14.7 (14.1, 15.4)
**ADHD**	892	1 192 643	2.7 (2.3, 3.0)	4863	6 122 704	13.6 (12.9, 14.3)
**Behavioral Problems**	355	533 986	1.2 (1.0, 1.5)	3410	4 596 562	10.2 (9.6, 10.9)
**Learning Disability**	303	397 489	0.9 (0.7, 1.1)	3037	4 173 686	9.3 (8.6, 9.9)
**Speech Disorder**	830	1 218 674	2.7 (2.4, 3.1)	2938	4 148 568	9.2 (8.6, 9.8)
**Depression**	226	355 961	0.8 (0.6, 1.1)	2616	2 997 482	6.7 (6.2, 7.2)
**DD/ID**	127	232 791	0.5 (0.3, 0.8)	2450	3 528 625	7.8 (7.2, 8.5)
**ASD**	65	94 738	0.2 (0.1, 0.3)	1424	2 000 776	4.4 (4.0, 4.9)
**Tourette Syndrome**	27	37 463	0.1 (0.0, 0.2)	130	188 133	0.4 (0.3, 0.6)

ADHD = Attention Deficit/Hyperactivity Disorder; DD/ID = Developmental Delay/Intellectual Disability; ASD = Autism Spectrum Disorder.
Weighted numbers and prevalence were calculated using survey sampling weights to represent the general population of US children aged 6–17 years. Unweighted numbers are presented as the observed number of cases.

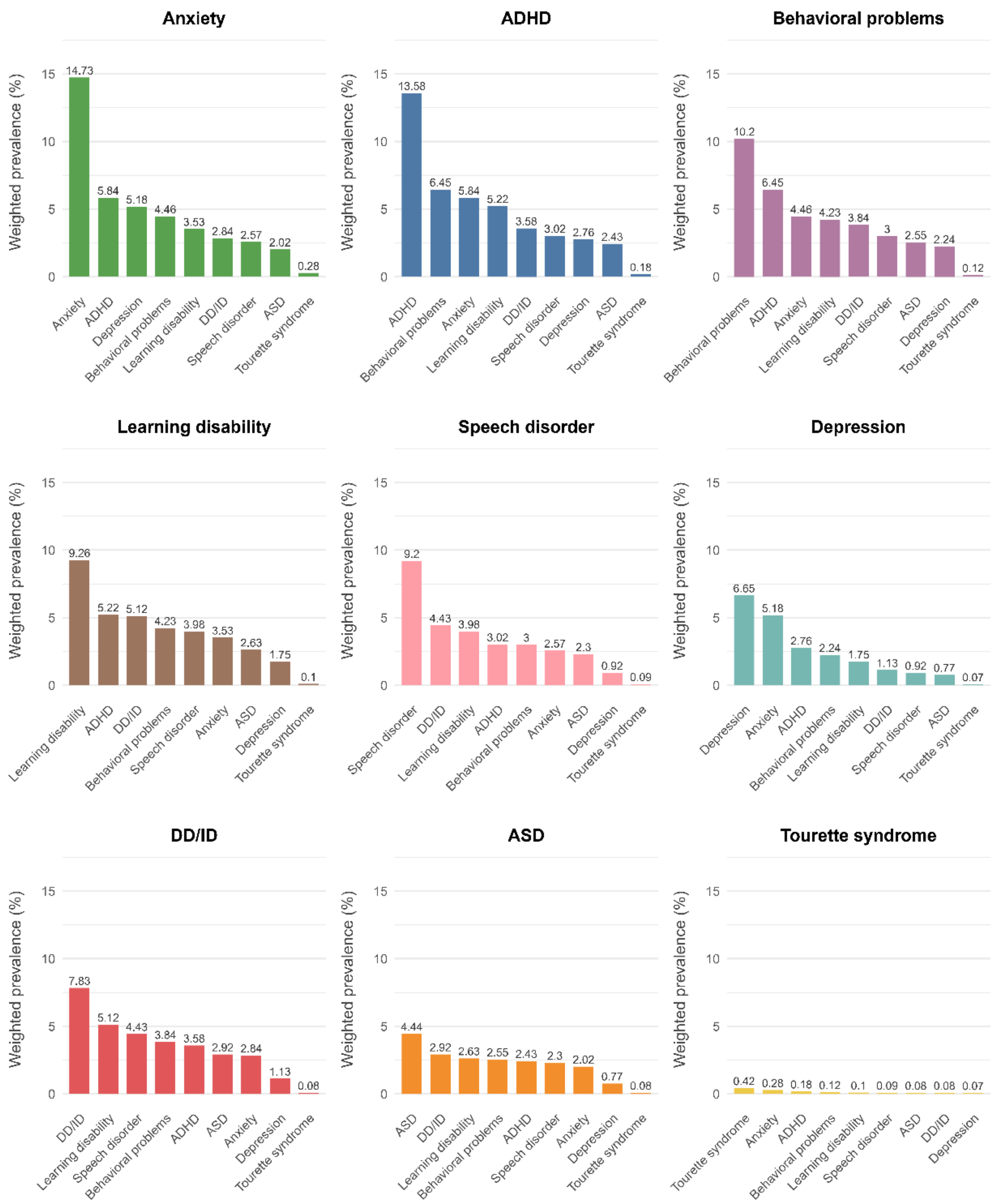

Figure S1. Weighted prevalence of individual mental health problems and their pairwise comorbidity combinations in US children aged 6–17 years.

ADHD = Attention Deficit/Hyperactivity Disorder; DD/ID = Developmental Delay/Intellectual Disability; ASD = Autism Spectrum Disorder.

Weighted prevalence were calculated using survey sampling weights to represent the general population of US children aged 6–17 years.

Table S4. Unweighted number and weighted prevalence (%) of individual mental health conditions and their pairwise comorbidity combinations in US children aged 6–17 years.

Mental health problems	ADHD	ASD	DD/D	Depression	Anxiety	Tourette syndrome	Behavioral problems	Speech disorder	Learning disability
**ADHD**	4863,13.6 (12.9, 14.3)	780, 2.4 (2.1, 2.9)	1170, 3.6 (3.2, 4.0)	1178, 2.8 (2.5, 3.1)	2352, 5.8 (5.4, 6.3)	69, 0.2 (0.1, 0.3)	2245, 6.5 (5.9, 7.0)	984, 3.0 (2.6, 3.5)	1743, 5.2 (4.7, 5.8)
**ASD**	780, 2.4 (2.1, 2.9)	1424, 4.4 (4.0, 4.9)	884, 2.9 (2.5, 3.4)	303, 0.8 (0.6, 0.9)	729, 2.0 (1.8, 2.3)	26, 0.1 (0.0, 0.2)	790, 2.6 (2.2, 3.0)	686, 2.3 (1.9, 2.7)	773, 2.6 (2.3, 3.1)
**DD/ID**	1170, 3.6 (3.2, 4.0)	884, 2.9 (2.5, 3.4)	2450, 7.8 (7.2, 8.5)	441, 1.1 (1.0 1.3)	1019, 2.8 (2.5, 3.2)	28, 0.1 (0.0, 0.2)	1188, 3.8 (3.4, 4.3)	1383, 4.4 (4.0, 4.9)	1586, 5.1 (4.6, 5.6)
**Depression**	1178, 2.8 (2.5, 3.1)	303, 0.8 (0.6, 0.9)	441, 1.1 (1.0, 1.3)	2616, 6.7 (6.2, 7.2)	2195, 5.2 (4.8, 5.6)	28, 0.1 (0.0, 0.1)	851, 2.2 (2.0, 2.6)	355, 0.9 (0.8, 1.1)	644, 1.8 (1.5, 2.1)
**Anxiety**	2352, 5.8 (5.4, 6.3)	729, 2.0 (1.8, 2.3)	1019, 2.8 (2.5, 3.2)	2195, 5.2 (4.8, 5.6)	5742, 14.7 (14.1, 15.4)	80, 0.3 (0.2, 0.5)	1684, 4.5 (4.1, 4.9)	919, 2.6 (2.3, 2.9)	1325, 3.5 (3.2, 3.9)
**Tourette syndrome**	69, 0.2 (0.1, 0.3)	26, 0.1 (0.0, 0.2)	28, 0.1 (0.0, 0.2)	28, 0.1 (0.0, 0.1)	80, 0.28 (0.2, 0.5)	130, 0.4 (0.3, 0.6)	36, 0.1 (0.1, 0.2)	25, 0.1 (0.0, 0.2)	45, 0.1 (0.1, 0.2)
**Behavioral problems**	2245, 6.5 (5.9, 7.0)	790, 2.6 (2.2, 3.0)	1188, 3.8 (3.4, 4.3)	851, 2.2 (2.0, 2.6)	1684, 4.5 (4.1, 4.9)	36, 0.1 (0.1, 0.2)	3410, 10.2 (9.6, 10.9)	942, 3.0 (2.6, 3.4)	1305, 4.2 (3.8, 4.8)
**Speech disorder**	984, 3.0 (2.6, 3.5)	686, 2.3 (1.9, 2.7)	1383, 4.4 (4.0, 4.9)	355, 0.9 (0.8, 1.1)	919, 2.6 (2.3, 2.9)	25, 0.1 (0.0, 0.2)	942, 3.0 (2.6, 3.4)	2938, 9.2 (8.6, 9.8)	1231, 4.0 (3.6, 4.5)
**Learning disability**	1743, 5.2 (4.7, 5.8)	773, 2.6 (2.3, 3.1)	1586, 5.1 (4.6, 5.6)	644, 1.8 (1.5, 2.1)	1325, 3.5 (3.2, 3.9)	45, 0.1 (0.1, 0.2)	1305, 4.2 (3.8, 4.8)	1231, 4.0 (3.6, 4.5)	3037, 9.3 (8.6, 9.9)

ADHD = Attention Deficit/Hyperactivity Disorder; ASD = Autism Spectrum Disorder; DD/ID = Developmental Delay/Intellectual Disability.

All cells present the unweighted number and weighted prevalence (%) (95% CI). Diagonal cells show these values for each single condition, irrespective of additional comorbidities among the nine mental health conditions. Off-diagonal cells show these values for children with both specified conditions, regardless of

additional comorbidities among the nine mental health conditions. All weighted prevalence estimates use the total weighted population of U.S. children aged 6–17 years as the denominator; numerators are weighted counts for the single condition (diagonal) or co-occurring conditions (off-diagonal), respectively.

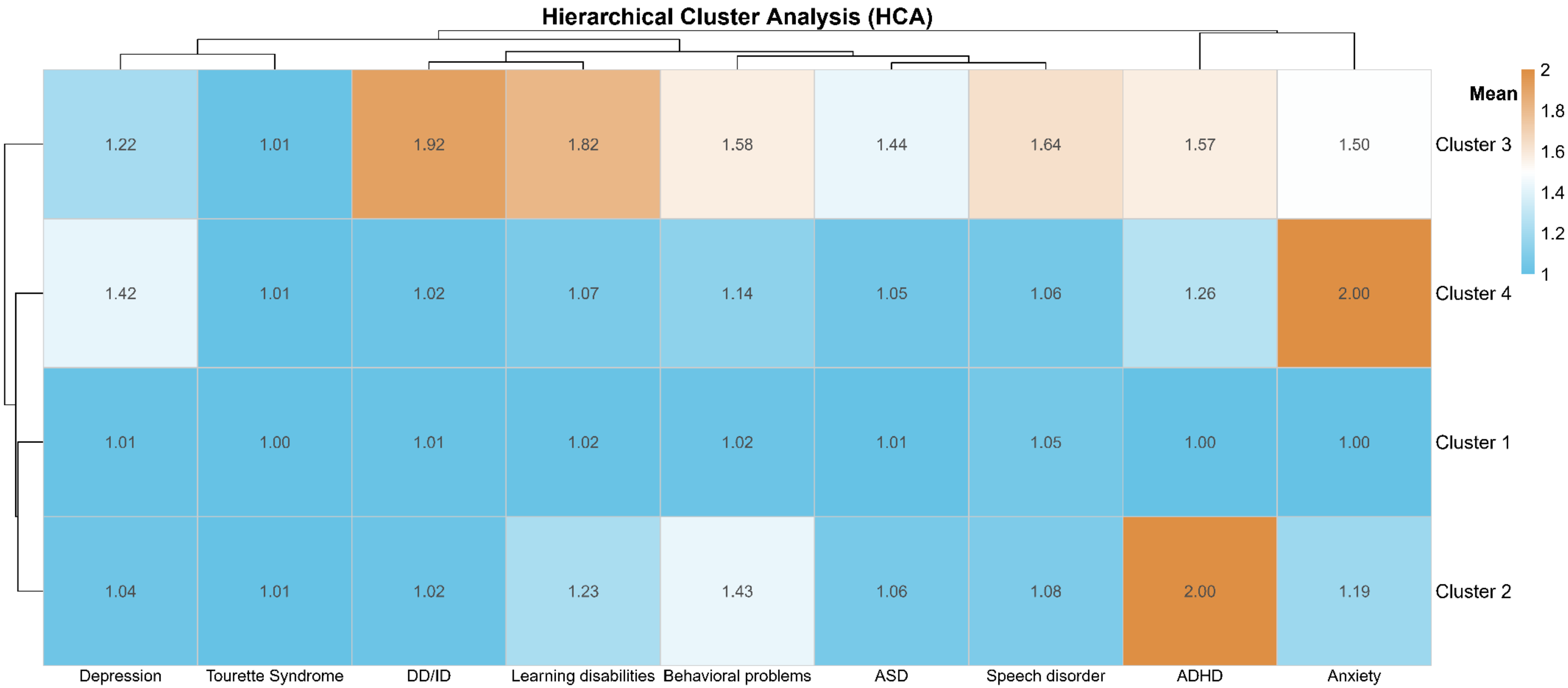

Figure S2. Hierarchical Cluster Analysis Heatmap of Nine Mental Health Problems.

ADHD = Attention Deficit/Hyperactivity Disorder; ASD = Autism Spectrum Disorder; DD/ID = Developmental Delay/Intellectual Disability.

Table S5. Distribution of mental health problems across Hierarchical Cluster Analysis (HCA) derived clusters.

Mental health problems	Cluster 1	Cluster 2	Cluster 3	Cluster 4
**ADHD**	0 (0.0%)	2540 (100.0%)	1223 (57.4%)	1100 (26.1%)
**ASD**	139 (0.6%)	159 (6.3%)	932 (43.7%)	194 (4.6%)
**DD/ID**	335 (1.5%)	60 (2.4%)	1956 (91.8%)	99 (2.4%)
**Depression**	275 (1.3%)	101 (4.0%)	461 (21.6%)	1779 (42.3%)
**Anxiety**	0 (0.0%)	477 (18.8%)	1058 (49.6%)	4207 (100.0%)
**Tourette syndrome**	33 (0.2%)	25 (1.0%)	30 (1.4%)	42 (1.0%)
**Behavioral problems**	483 (2.2%)	1102 (43.4%)	1235 (58.0%)	590 (14.0%)
**Speech disorder**	1118 (5.2%)	213 (8.4%)	1358 (63.7%)	249 (5.9%)
**Learning disability**	409 (1.9%)	576 (22.7%)	1744 (81.8%)	308 (7.3%)

All values in the table represent the unweighted number of cases followed by the percentage of the respective mental health problem within each cluster in parentheses (i.e., “number (%)”). The table presents the distribution of nine mental health problems across four clusters identified through hierarchical cluster analysis (HCA).

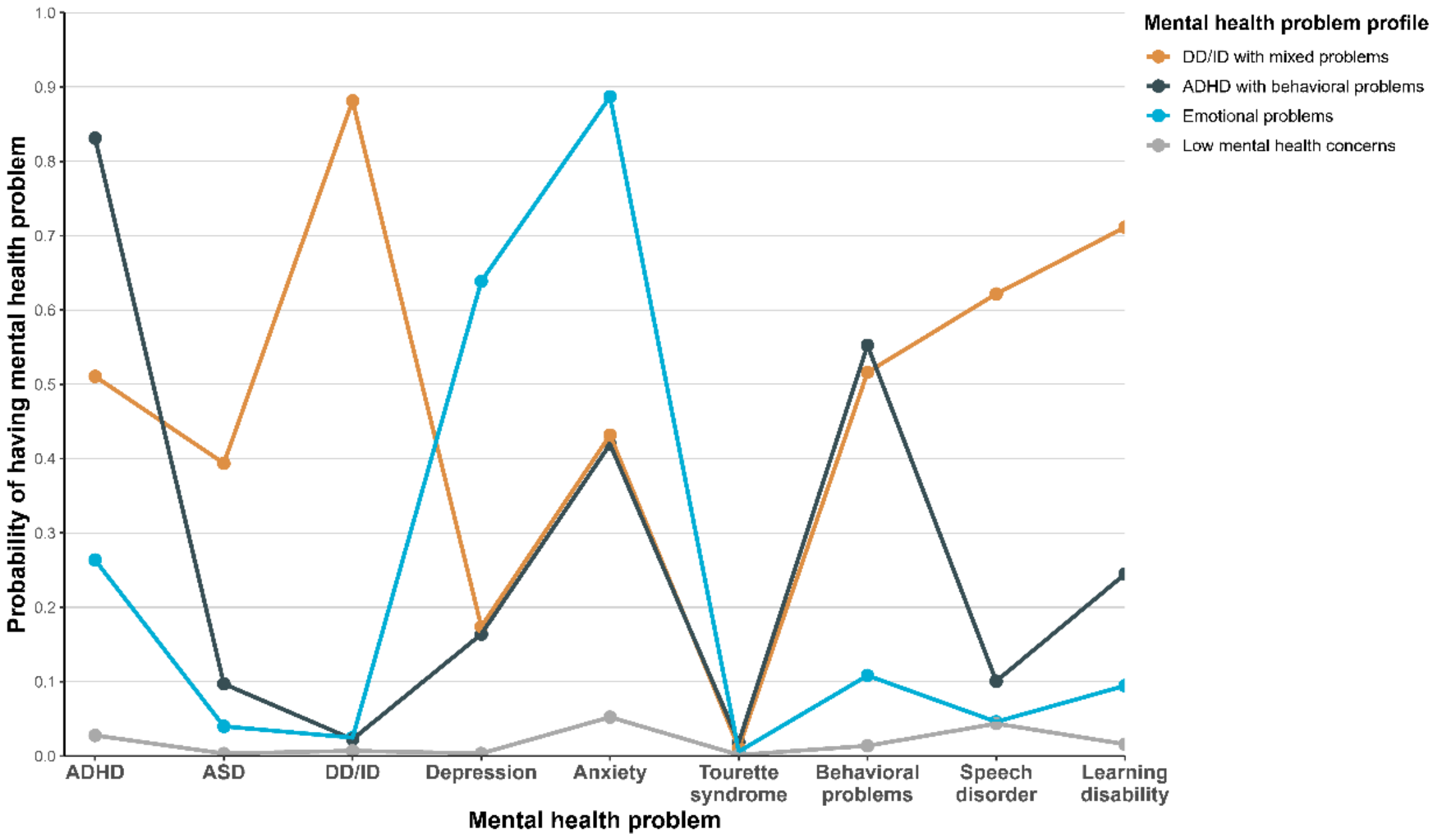

Figure S3. Profiles of mental health multimorbidity among US children aged 6-17 years identified through latent class analysis model after multiple imputation for variables with missing records related to mental health problems or physical conditions.

ADHD = Attention Deficit/Hyperactivity Disorder; ASD = Autism Spectrum Disorder; DD/ID = Developmental Delay/Intellectual Disability.

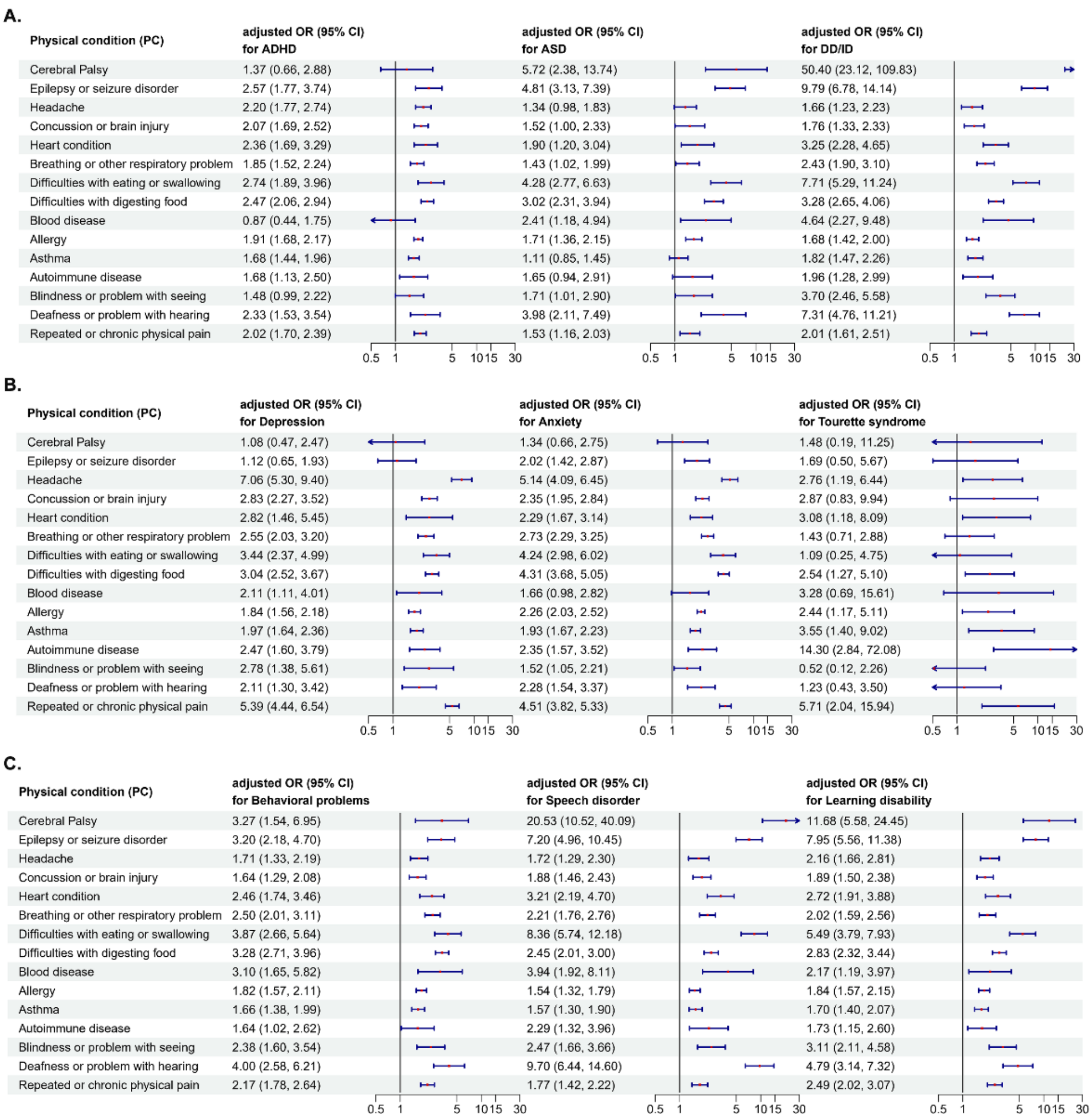

Figure S4. Associations of specific mental health problem with physical conditions in US children aged 6-17 years (weighted for complex survey design).

OR = Odds Ratio; CI = Confidence Interval. ADHD = Attention Deficit/Hyperactivity Disorder; ASD = Autism Spectrum Disorder; DD/ID = Developmental Delay/Intellectual Disability. Logistic regression models were weighted to account for the complex survey design of the NSCH, including strata, clusters, and sampling weights, and adjusted for children's characteristics (age, sex, gestational age, Race and Ethnicity) and family characteristics (education level, marital status and family income).

*Diagnosed within the past 12 months.

Physical condition (PC)	Mental health problem profile	No. of PC	adjusted OR (95% CI)
Cerebral Palsy	DD/ID with Mixed Problems	26	66.87 (23.92, 186.94)
	Emotional Problems	3	1.00 (0.15, 6.46)
	ADHD with Behavioral Problems	–	–
Epilepsy or seizure disorder	DD/ID with Mixed Problems	67	16.21 (8.87, 29.64)
	Emotional Problems	32	1.48 (0.73, 3.01)
	ADHD with Behavioral Problems	8	2.73 (0.85, 8.79)
Headache	DD/ID with Mixed Problems	90	3.18 (1.87, 5.42)
	Emotional Problems	341	4.67 (3.03, 7.22)
	ADHD with Behavioral Problems	44	2.74 (1.62, 4.64)
Concussion or brain injury	DD/ID with Mixed Problems	60	1.62 (0.84, 3.13)
	Emotional Problems	263	1.59 (1.14, 2.21)
	ADHD with Behavioral Problems	39	3.26 (1.66, 6.41)
Heart condition	DD/ID with Mixed Problems	65	5.41 (3.12, 9.38)
	Emotional Problems	80	5.81 (2.16, 15.65)
	ADHD with Behavioral Problems	23	5.40 (2.15, 13.55)
Breathing or other respiratory problem	DD/ID with Mixed Problems	86	2.52 (1.49, 4.27)
	Emotional Problems	239	3.12 (2.22, 4.40)
	ADHD with Behavioral Problems	54	1.48 (0.91, 2.42)
Difficulties with eating or swallowing	DD/ID with Mixed Problems	63	15.73 (7.88, 31.42)
	Emotional Problems	70	5.44 (3.03, 9.75)
	ADHD with Behavioral Problems	11	3.57 (1.46, 8.72)
Difficulties with digesting food	DD/ID with Mixed Problems	161	4.04 (2.72, 5.99)
	Emotional Problems	363	3.89 (2.94, 5.14)
	ADHD with Behavioral Problems	84	3.70 (2.34, 5.84)
Blood disease	DD/ID with Mixed Problems	14	6.38 (1.76, 23.08)
	Emotional Problems	13	1.20 (0.40, 3.60)
	ADHD with Behavioral Problems	2	0.96 (0.22, 4.23)
Allergy	DD/ID with Mixed Problems	315	1.80 (1.31, 2.48)
	Emotional Problems	925	1.89 (1.54, 2.32)
	ADHD with Behavioral Problems	221	1.71 (1.25, 2.35)
Asthma	DD/ID with Mixed Problems	106	1.47 (0.94, 2.31)
	Emotional Problems	389	1.83 (1.38, 2.41)
	ADHD with Behavioral Problems	68	1.45 (0.93, 2.26)
Autoimmune disease	DD/ID with Mixed Problems	26	2.58 (1.14, 5.79)
	Emotional Problems	78	1.51 (0.74, 3.06)
	ADHD with Behavioral Problems	10	2.27 (0.99, 5.23)
Blindness or problem with seeing	DD/ID with Mixed Problems	56	6.35 (3.29, 12.26)
	Emotional Problems	39	2.82 (0.70, 11.31)
	ADHD with Behavioral Problems	13	1.67 (0.71, 3.90)
Deafness or problem with hearing	DD/ID with Mixed Problems	53	14.51 (7.01, 30.03)
	Emotional Problems	28	3.65 (1.79, 7.44)
	ADHD with Behavioral Problems	9	1.24 (0.46, 3.29)
Repeated or chronic physical pain	DD/ID with Mixed Problems	152	3.07 (2.07, 4.55)
	Emotional Problems	472	2.79 (2.16, 3.61)
	ADHD with Behavioral Problems	73	2.29 (1.50, 3.49)

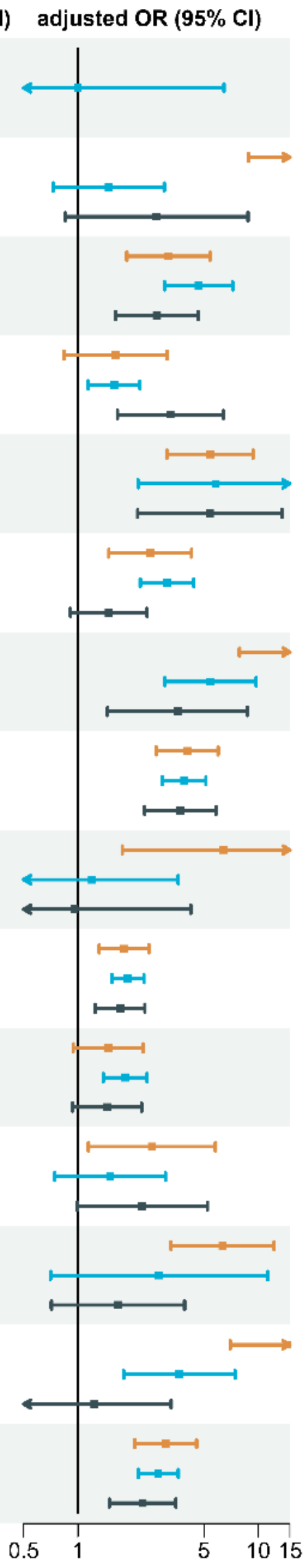

Figure S5. Associations of profiles of mental health multimorbidity with physical conditions in US girls aged 6-17 years (weighted for complex survey design).

OR = Odds Ratio; CI = Confidence Interval. ADHD = Attention Deficit/Hyperactivity Disorder; DD/ID = Developmental Delay/Intellectual Disability.

Results were based on joint modeling of latent class analysis of mental health problems and logistic regression of physical conditions on the class membership. Logistic regression models were weighted to account for the complex survey design of the NSCH, including strata, clusters, and sampling weights, and adjusted for children’s characteristics (age, sex, gestational age, Race and Ethnicity) and family characteristics (education level, marital status and family income).

*Diagnosed within the past 12 months.

Physical condition (PC)	Mental health problem profile	No. of PC	adjusted OR (95%CI)
Cerebral Palsy	DD/ID with Mixed Problems	37	16.88 (6.40, 44.48)
	Emotional Problems	–	–
	ADHD with Behavioral Problems	2	0.54 (0.08, 3.44)
Epilepsy or seizure disorder	DD/ID with Mixed Problems	84	8.96 (5.16, 15.53)
	Emotional Problems	2	0.12 (0.02, 0.63)
	ADHD with Behavioral Problems	28	2.16 (1.02, 4.59)
Headache	DD/ID with Mixed Problems	118	2.56 (1.79, 3.66)
	Emotional Problems	78	7.20 (4.44, 11.68)
	ADHD with Behavioral Problems	124	2.27 (1.54, 3.36)
Concussion or brain injury	DD/ID with Mixed Problems	164	2.25 (1.61, 3.14)
	Emotional Problems	97	3.53 (2.32, 5.38)
	ADHD with Behavioral Problems	209	1.67 (1.28, 2.17)
Heart condition	DD/ID with Mixed Problems	110	3.23 (2.02, 5.15)
	Emotional Problems	14	1.69 (0.72, 3.94)
	ADHD with Behavioral Problems	68	1.73 (1.11, 2.67)
Breathing or other respiratory problem	DD/ID with Mixed Problems	229	2.29 (1.72, 3.07)
	Emotional Problems	44	2.10 (1.17, 3.79)
	ADHD with Behavioral Problems	184	1.87 (1.40, 2.49)
Difficulties with eating or swallowing	DD/ID with Mixed Problems	101	7.00 (4.20, 11.64)
	Emotional Problems	17	4.01 (1.86, 8.64)
	ADHD with Behavioral Problems	19	2.13 (0.96, 4.70)
Difficulties with digesting food	DD/ID with Mixed Problems	323	4.21 (3.19, 5.55)
	Emotional Problems	64	1.94 (1.25, 3.00)
	ADHD with Behavioral Problems	224	2.46 (1.91, 3.19)
Blood disease	DD/ID with Mixed Problems	16	2.79 (1.20, 6.44)
	Emotional Problems	4	4.04 (1.14, 14.29)
	ADHD with Behavioral Problems	6	0.99 (0.27, 3.58)
Allergy	DD/ID with Mixed Problems	728	1.77 (1.44, 2.17)
	Emotional Problems	212	1.89 (1.36, 2.63)
	ADHD with Behavioral Problems	808	1.63 (1.33, 1.99)
Asthma	DD/ID with Mixed Problems	331	1.68 (1.32, 2.16)
	Emotional Problems	93	1.38 (0.95, 1.99)
	ADHD with Behavioral Problems	320	1.43 (1.12, 1.83)
Autoimmune disease	DD/ID with Mixed Problems	40	2.10 (1.26, 3.51)
	Emotional Problems	12	1.03 (0.47, 2.26)
	ADHD with Behavioral Problems	35	1.85 (0.91, 3.75)
Blindness or problem with seeing	DD/ID with Mixed Problems	79	3.47 (1.93, 6.26)
	Emotional Problems	9	0.52 (0.14, 1.88)
	ADHD with Behavioral Problems	31	1.54 (0.76, 3.13)
Deafness or problem with hearing	DD/ID with Mixed Problems	88	6.03 (3.58, 10.15)
	Emotional Problems	9	1.01 (0.40, 2.52)
	ADHD with Behavioral Problems	21	2.34 (1.11, 4.94)
Repeated or chronic physical pain	DD/ID with Mixed Problems	196	2.78 (2.08, 3.72)
	Emotional Problems	82	4.52 (2.94, 6.97)
	ADHD with Behavioral Problems	188	2.67 (1.95, 3.64)

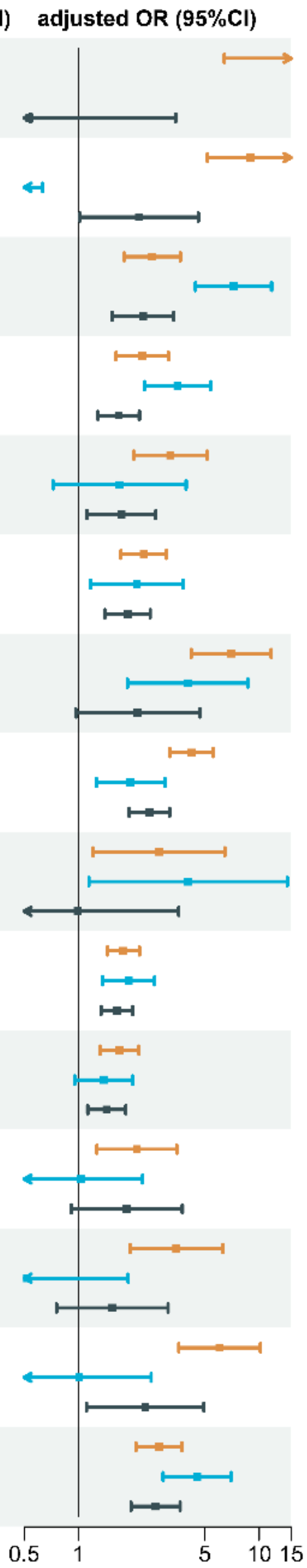

Figure S6. Associations of profiles of mental health multimorbidity with physical conditions in US boys aged 6-17 years (weighted for complex survey design).

OR = Odds Ratio; CI = Confidence Interval. ADHD = Attention Deficit/Hyperactivity Disorder; DD/ID = Developmental Delay/Intellectual Disability.

Results were based on joint modeling of latent class analysis of mental health problems and logistic regression of physical conditions on the class membership. Logistic regression models were weighted to account for the complex survey design of the NSCH, including strata, clusters, and sampling weights, and adjusted for children's characteristics (age, sex, gestational age, Race and Ethnicity) and family characteristics (education level, marital status and family income).

*Diagnosed within the past 12 months.

Physical condition (PC)	Mental health problem profile	No. of PC	adjusted OR (95% CI)
Cerebral Palsy	DD/ID with Mixed Problems	67	36.72 (16.45, 81.96)
	Emotional Problems	3	0.56 (0.10, 3.28)
	ADHD with Behavioral Problems	3	1.60 (0.34, 7.44)
Epilepsy or seizure disorder	DD/ID with Mixed Problems	165	11.39 (7.54, 17.21)
	Emotional Problems	35	1.20 (0.63, 2.29)
	ADHD with Behavioral Problems	37	2.22 (1.21, 4.10)
Headache	DD/ID with Mixed Problems	226	2.80 (2.07, 3.79)
	Emotional Problems	456	4.80 (3.38, 6.83)
	ADHD with Behavioral Problems	189	2.38 (1.75, 3.23)
Concussion or brain injury	DD/ID with Mixed Problems	240	2.17 (1.62, 2.91)
	Emotional Problems	387	1.99 (1.53, 2.58)
	ADHD with Behavioral Problems	274	1.89 (1.43, 2.50)
Heart condition	DD/ID with Mixed Problems	196	4.18 (2.94, 5.95)
	Emotional Problems	97	3.55 (1.56, 8.10)
	ADHD with Behavioral Problems	100	2.60 (1.69, 4.01)
Breathing or other respiratory problem	DD/ID with Mixed Problems	361	2.58 (2.02, 3.29)
	Emotional Problems	313	3.02 (2.31, 3.94)
	ADHD with Behavioral Problems	266	1.79 (1.42, 2.26)
Difficulties with eating or swallowing	DD/ID with Mixed Problems	181	10.34 (6.66, 16.05)
	Emotional Problems	98	4.69 (3.11, 7.07)
	ADHD with Behavioral Problems	34	2.87 (1.59, 5.18)
Difficulties with digesting food	DD/ID with Mixed Problems	523	4.13 (3.32, 5.13)
	Emotional Problems	469	3.26 (2.61, 4.06)
	ADHD with Behavioral Problems	331	2.74 (2.17, 3.45)
Blood disease	DD/ID with Mixed Problems	36	3.66 (1.69, 7.91)
	Emotional Problems	16	1.28 (0.53, 3.12)
	ADHD with Behavioral Problems	14	1.09 (0.48, 2.50)
Allergy	DD/ID with Mixed Problems	1143	1.76 (1.49, 2.08)
	Emotional Problems	1232	1.91 (1.61, 2.27)
	ADHD with Behavioral Problems	1109	1.63 (1.39, 1.91)
Asthma	DD/ID with Mixed Problems	481	1.67 (1.36, 2.04)
	Emotional Problems	521	1.66 (1.35, 2.03)
	ADHD with Behavioral Problems	424	1.49 (1.21, 1.82)
Autoimmune disease	DD/ID with Mixed Problems	74	2.38 (1.49, 3.81)
	Emotional Problems	95	1.36 (0.80, 2.32)
	ADHD with Behavioral Problems	51	2.01 (1.19, 3.42)
Blindness or problem with seeing	DD/ID with Mixed Problems	151	4.43 (2.89, 6.80)
	Emotional Problems	53	2.04 (0.66, 6.27)
	ADHD with Behavioral Problems	48	1.72 (0.96, 3.06)
Deafness or problem with hearing	DD/ID with Mixed Problems	154	7.80 (4.86, 12.52)
	Emotional Problems	39	1.94 (1.11, 3.39)
	ADHD with Behavioral Problems	31	2.23 (1.16, 4.27)
Repeated or chronic physical pain	DD/ID with Mixed Problems	381	2.82 (2.23, 3.56)
	Emotional Problems	600	2.99 (2.40, 3.71)
	ADHD with Behavioral Problems	287	2.40 (1.87, 3.08)

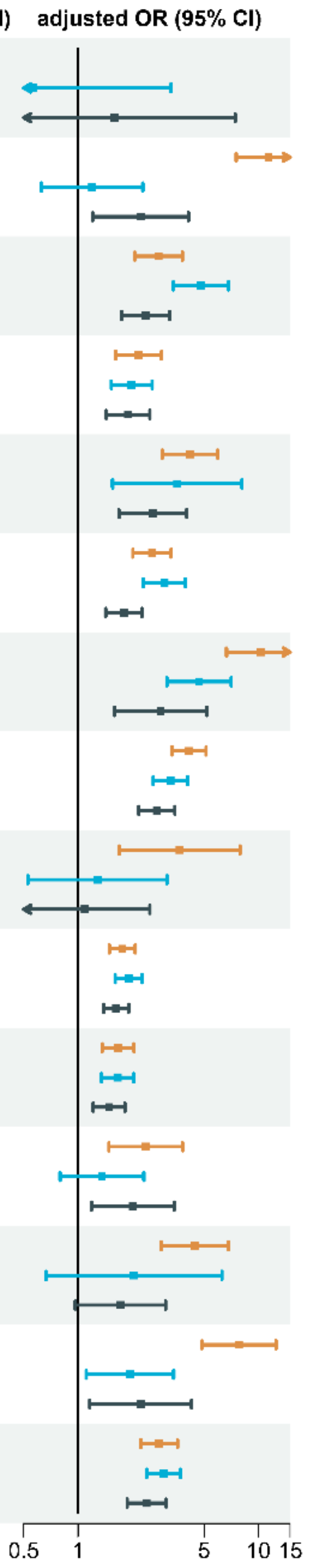

Figure S7. Associations of profiles of mental health multimorbidity with physical conditions in US children aged 6-17 years after multiple imputation for variables with missing records related to mental health problems or physical conditions (weighted for complex survey design).

OR = Odds Ratio; CI = Confidence Interval. ADHD = Attention Deficit/Hyperactivity Disorder; DD/ID = Developmental Delay/Intellectual Disability.

Results were based on joint modeling of latent class analysis of mental health problems and logistic regression of physical conditions on the class membership. Logistic regression models were weighted to account for the complex survey design of the NSCH, including strata, clusters, and sampling weights, and adjusted for children's characteristics (age, sex, gestational age, Race and Ethnicity) and family characteristics (education level, marital status and family income).

*Diagnosed within the past 12 months.

Physical condition (PC)	No. of comorbid mental health problems	No. of PC	adjusted OR (95% CI)
Cerebral Palsy	> 3	15	97.40 (25.27, 375.41)
	3	11	32.17 (7.82, 132.29)
	2	3	25.58 (3.21, 203.76)
	1	7	9.71 (2.43, 38.78)
Epilepsy or seizure disorder	> 3	45	12.03 (6.04, 23.97)
	3	28	5.73 (2.82, 11.63)
	2	30	5.50 (2.36, 12.81)
	1	26	1.79 (0.89, 3.60)
Headache	> 3	135	5.34 (3.35, 8.51)
	3	87	3.36 (2.13, 5.29)
	2	172	3.59 (2.43, 5.31)
	1	179	3.12 (1.86, 5.21)
Concussion or brain injury	> 3	87	2.44 (1.32, 4.50)
	3	64	1.52 (0.92, 2.53)
	2	139	2.10 (1.36, 3.24)
	1	172	1.49 (1.06, 2.09)
Heart condition	> 3	58	7.87 (4.43, 14.00)
	3	39	8.37 (3.66, 19.14)
	2	49	4.66 (2.28, 9.50)
	1	75	6.70 (3.08, 14.56)
Breathing or other respiratory problem	> 3	103	3.65 (2.19, 6.06)
	3	72	2.13 (1.23, 3.68)
	2	164	3.86 (2.67, 5.57)
	1	182	3.26 (2.25, 4.72)
Difficulties with eating or swallowing	> 3	61	31.45 (14.71, 67.25)
	3	32	10.31 (5.04, 21.10)
	2	43	10.53 (4.80, 23.12)
	1	38	6.25 (2.82, 13.81)
Difficulties with digesting food	> 3	205	6.48 (4.46, 9.43)
	3	125	3.89 (2.65, 5.71)
	2	213	4.14 (3.03, 5.65)
	1	261	2.42 (1.83, 3.21)
Blood disease	> 3	13	3.55 (1.31, 9.61)
	3	2	0.56 (0.10, 3.12)
	2	13	6.11 (1.92, 19.49)
	1	11	2.04 (0.48, 8.68)
Allergy	> 3	367	2.57 (1.86, 3.54)
	3	328	1.78 (1.33, 2.37)
	2	577	2.02 (1.61, 2.54)
	1	849	1.73 (1.43, 2.10)
Asthma	> 3	136	1.86 (1.20, 2.88)
	3	127	1.70 (1.22, 2.38)
	2	231	2.55 (1.83, 3.57)
	1	274	2.02 (1.49, 2.73)
Autoimmune disease	> 3	28	3.12 (1.28, 7.59)
	3	23	1.40 (0.63, 3.13)
	2	50	3.70 (1.38, 9.88)
	1	51	2.21 (0.93, 5.24)
Blindness or problem with seeing	> 3	49	6.54 (3.28, 13.03)
	3	24	1.70 (0.76, 3.77)
	2	24	0.85 (0.40, 1.82)
	1	36	2.53 (0.78, 8.19)
Deafness or problem with hearing	> 3	42	24.64 (10.76, 56.43)
	3	24	6.22 (2.85, 13.55)
	2	20	2.50 (1.14, 5.47)
	1	28	5.17 (2.35, 11.35)
Repeated or chronic physical pain	> 3	218	5.33 (3.72, 7.63)
	3	131	2.98 (2.13, 4.16)
	2	257	3.36 (2.39, 4.72)
	1	260	2.21 (1.64, 2.98)

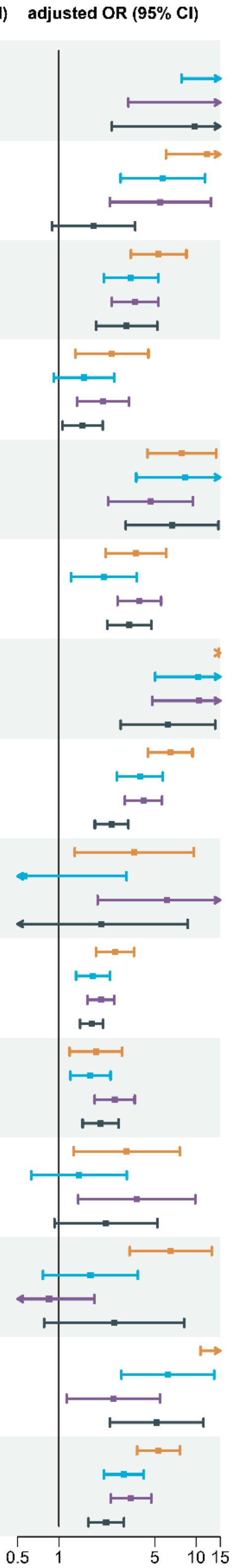

Figure S8. Associations between numbers of mental health multimorbidity and physical conditions in US girls aged 6-17 years (weighted for complex survey design).

OR = Odds Ratio; CI = Confidence Interval.

Logistic regression models were weighted to account for the complex survey design of the NSCH,

including strata, clusters, and sampling weights, and adjusted for children's characteristics (age, sex, gestational age, Race and Ethnicity) and family characteristics (education level, marital status and family income).
*Diagnosed within the past 12 months.

Physical condition (PC)	No. of comorbid mental health problems	No. of PC	adjusted OR (95% CI)
Cerebral Palsy	> 3	20	11.92 (3.11, 45.73)
	3	13	17.68 (4.10, 76.25)
	2	6	4.25 (0.90, 20.08)
	1	7	2.83 (0.58, 13.68)
Epilepsy or seizure disorder	> 3	67	8.05 (4.32, 15.00)
	3	29	6.26 (2.95, 13.29)
	2	18	2.05 (0.80, 5.25)
	1	20	1.41 (0.63, 3.14)
Headache	> 3	158	4.50 (3.09, 6.57)
	3	72	2.87 (1.76, 4.69)
	2	84	2.20 (1.42, 3.40)
	1	119	1.62 (1.08, 2.42)
Concussion or brain injury	> 3	173	2.63 (1.88, 3.68)
	3	105	2.86 (2.01, 4.06)
	2	177	2.28 (1.65, 3.16)
	1	246	2.11 (1.61, 2.77)
Heart condition	> 3	100	3.01 (1.90, 4.77)
	3	44	2.96 (1.40, 6.30)
	2	44	1.32 (0.75, 2.33)
	1	69	1.06 (0.65, 1.72)
Breathing or other respiratory problem	> 3	224	3.09 (2.29, 4.17)
	3	93	1.88 (1.30, 2.71)
	2	133	1.99 (1.42, 2.77)
	1	195	1.64 (1.21, 2.23)
Difficulties with eating or swallowing	> 3	85	9.41 (5.42, 16.34)
	3	26	4.35 (2.24, 8.45)
	2	24	3.54 (1.65, 7.62)
	1	29	1.93 (1.00, 3.72)
Difficulties with digesting food	> 3	342	6.57 (4.93, 8.76)
	3	121	3.14 (2.27, 4.34)
	2	149	2.51 (1.83, 3.45)
	1	200	2.34 (1.73, 3.16)
Blood disease	> 3	14	2.88 (1.16, 7.16)
	3	8	2.28 (0.83, 6.23)
	2	3	1.14 (0.24, 5.33)
	1	10	1.37 (0.42, 4.49)
Allergy	> 3	732	2.46 (1.97, 3.08)
	3	404	1.79 (1.42, 2.26)
	2	586	1.40 (1.14, 1.73)
	1	927	1.45 (1.22, 1.72)
Asthma	> 3	332	1.98 (1.54, 2.56)
	3	154	1.58 (1.13, 2.20)
	2	236	1.34 (1.02, 1.76)
	1	367	1.46 (1.16, 1.85)
Autoimmune disease	> 3	39	3.12 (1.80, 5.40)
	3	23	1.94 (0.96, 3.92)
	2	22	1.83 (0.74, 4.54)
	1	38	2.27 (1.25, 4.14)
Blindness or problem with seeing	> 3	61	3.14 (1.62, 6.10)
	3	30	2.08 (1.02, 4.24)
	2	25	1.54 (0.73, 3.25)
	1	29	1.62 (0.74, 3.52)
Deafness or problem with hearing	> 3	68	5.18 (2.80, 9.58)
	3	33	5.49 (2.82, 10.66)
	2	18	3.86 (1.57, 9.49)
	1	35	1.90 (0.97, 3.73)
Repeated or chronic physical pain	> 3	235	4.89 (3.58, 6.68)
	3	102	3.51 (2.41, 5.13)
	2	122	3.34 (2.31, 4.82)
	1	184	2.87 (2.06, 3.98)

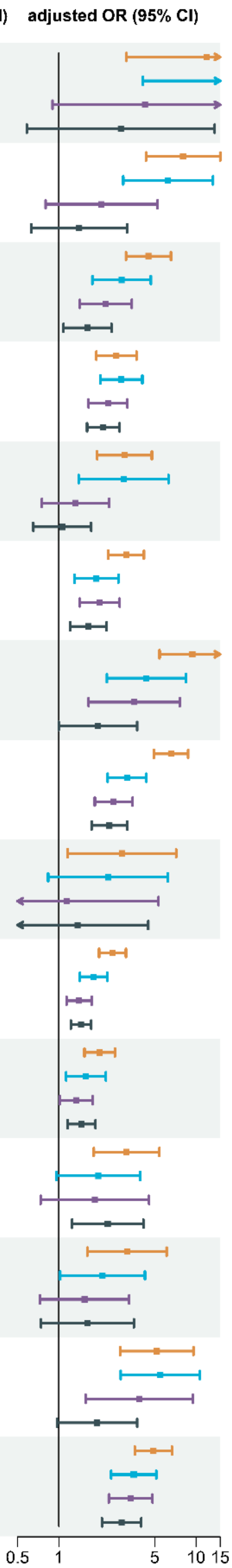

Figure S9. Associations between numbers of mental health multimorbidity and physical conditions in US boys aged 6-17 years (weighted for complex survey design).

OR = Odds Ratio; CI = Confidence Interval.

Logistic regression models were weighted to account for the complex survey design of the NSCH, including strata, clusters, and sampling weights, and adjusted for children's characteristics (age, sex, gestational age, Race and Ethnicity) and family characteristics (education level, marital status and family income).
*Diagnosed within the past 12 months.

Physical condition (PC)	No. of comorbid mental health problems	No. of PC	adjusted OR (95% CI)
Cerebral Palsy	> 3	36	30.27 (10.33, 88.72)
	3	26	23.77 (8.38, 67.41)
	2	11	11.51 (2.59, 51.17)
	1	14	4.57 (1.52, 13.72)
Epilepsy or seizure disorder	> 3	124	9.72 (6.13, 15.42)
	3	60	5.62 (3.38, 9.32)
	2	51	3.41 (1.78, 6.53)
	1	54	1.61 (0.98, 2.64)
Headache	> 3	318	4.85 (3.61, 6.53)
	3	184	3.18 (2.29, 4.42)
	2	277	3.13 (2.29, 4.29)
	1	333	2.40 (1.66, 3.45)
Concussion or brain injury	> 3	285	2.69 (2.01, 3.61)
	3	189	2.61 (1.92, 3.54)
	2	333	2.15 (1.66, 2.79)
	1	462	1.91 (1.53, 2.40)
Heart condition	> 3	170	4.36 (3.00, 6.34)
	3	92	5.07 (2.99, 8.60)
	2	105	2.35 (1.50, 3.67)
	1	153	2.89 (1.59, 5.24)
Breathing or other respiratory problem	> 3	369	3.66 (2.87, 4.68)
	3	187	2.02 (1.49, 2.74)
	2	336	2.85 (2.25, 3.61)
	1	426	2.30 (1.83, 2.89)
Difficulties with eating or swallowing	> 3	161	16.87 (10.89, 26.14)
	3	65	8.27 (4.91, 13.94)
	2	75	6.61 (3.94, 11.09)
	1	81	4.16 (2.34, 7.41)
Difficulties with digesting food	> 3	594	6.45 (5.18, 8.03)
	3	270	3.56 (2.77, 4.58)
	2	385	3.07 (2.46, 3.83)
	1	509	2.34 (1.91, 2.88)
Blood disease	> 3	32	2.89 (1.57, 5.31)
	3	11	1.24 (0.56, 2.74)
	2	20	2.63 (1.01, 6.87)
	1	24	1.36 (0.58, 3.18)
Allergy	> 3	1199	2.42 (2.04, 2.88)
	3	792	1.82 (1.52, 2.16)
	2	1261	1.72 (1.47, 2.00)
	1	1948	1.59 (1.40, 1.80)
Asthma	> 3	514	2.03 (1.66, 2.50)
	3	308	1.65 (1.30, 2.10)
	2	513	1.80 (1.47, 2.20)
	1	711	1.63 (1.36, 1.94)
Autoimmune disease	> 3	74	3.31 (2.02, 5.41)
	3	51	1.61 (0.96, 2.69)
	2	76	2.60 (1.34, 5.04)
	1	98	2.17 (1.24, 3.80)
Blindness or problem with seeing	> 3	121	4.26 (2.66, 6.82)
	3	62	2.08 (1.25, 3.47)
	2	58	1.39 (0.82, 2.37)
	1	68	1.85 (0.89, 3.87)
Deafness or problem with hearing	> 3	123	10.54 (6.15, 18.05)
	3	58	5.71 (3.42, 9.52)
	2	40	3.48 (1.69, 7.16)
	1	69	3.69 (2.04, 6.67)
Repeated or chronic physical pain	> 3	498	4.99 (3.97, 6.29)
	3	261	3.27 (2.55, 4.20)
	2	411	3.30 (2.59, 4.21)
	1	484	2.42 (1.93, 3.02)

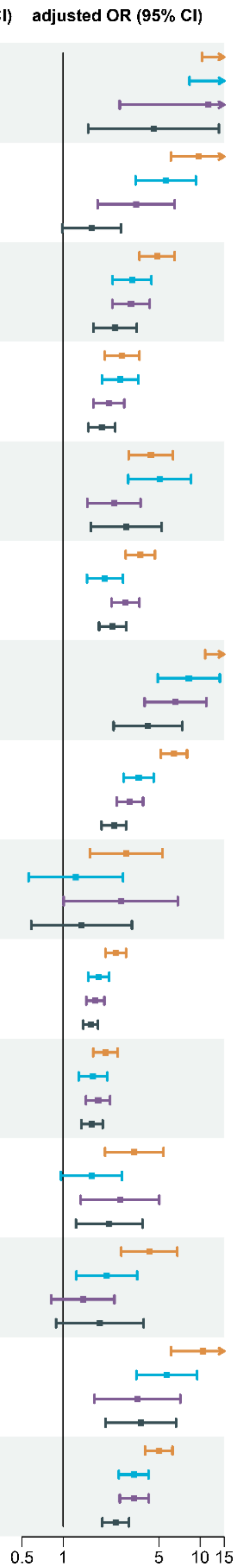

Figure S10. Associations between numbers of mental health multimorbidity and physical conditions in US children aged 6-17 years after multiple imputation for variables with missing records related to mental health problems or physical

conditions (weighted for complex survey design).

OR = Odds Ratio; CI = Confidence Interval.

Logistic regression models were weighted to account for the complex survey design of the NSCH, including strata, clusters, and sampling weights, and adjusted for children's characteristics (age, sex, gestational age, Race and Ethnicity) and family characteristics (education level, marital status and family income).

*Diagnosed within the past 12 months.
